# Data-driven support to decision-making in molecular tumour boards for lymphoma: A design science approach

**DOI:** 10.3389/fonc.2022.984021

**Published:** 2022-11-15

**Authors:** Núria Rodríguez Ruiz, Sulaf Abd Own, Karin Ekström Smedby, Sandra Eloranta, Sabine Koch, Tove Wästerlid, Aleksandra Krstic, Magnus Boman

**Affiliations:** ^1^ Department of Learning, Informatics, Management and Ethics (LIME), Health Informatics Centre, Karolinska Institutet, Stockholm, Sweden; ^2^ Department of Medicine Solna, Clinical Epidemiology Division, Karolinska Institutet, Stockholm, Sweden; ^3^ Department of Laboratory Medicine, Division of Pathology, Karolinska University Hospital Huddinge, Stockholm, Sweden; ^4^ Department of Hematology, Karolinska University Hospital, Stockholm, Sweden; ^5^ Center for Hematology and Regenerative Medicine, Karolinska Institutet, Stockholm, Sweden; ^6^ Department of Laboratory Medicine, Karolinska Institutet, Stockholm, Sweden; ^7^ School of Electrical Engineering and Computer Science (EECS)/Software and Computer Systems, KTH Royal Institute of Technology, Stockholm, Sweden

**Keywords:** precision medicine, next-generation sequencing, molecular tumour board, clinical decision support system, artificial intelligence, multimodal data, lymphoma

## Abstract

**Background:**

The increasing amount of molecular data and knowledge about genomic alterations from next-generation sequencing processes together allow for a greater understanding of individual patients, thereby advancing precision medicine. Molecular tumour boards feature multidisciplinary teams of clinical experts who meet to discuss complex individual cancer cases. Preparing the meetings is a manual and time-consuming process.

**Purpose:**

To design a clinical decision support system to improve the multimodal data interpretation in molecular tumour board meetings for lymphoma patients at Karolinska University Hospital, Stockholm, Sweden. We investigated user needs and system requirements, explored the employment of artificial intelligence, and evaluated the proposed design with primary stakeholders.

**Methods:**

Design science methodology was used to form and evaluate the proposed artefact. Requirements elicitation was done through a scoping review followed by five semi-structured interviews. We used UML Use Case diagrams to model user interaction and UML Activity diagrams to inform the proposed flow of control in the system. Additionally, we modelled the current and future workflow for MTB meetings and its proposed machine learning pipeline. Interactive sessions with end-users validated the initial requirements based on a fictive patient scenario which helped further refine the system.

**Results:**

The analysis showed that an interactive secure Web-based information system supporting the preparation of the meeting, multidisciplinary discussions, and clinical decision-making could address the identified requirements. Integrating artificial intelligence *via* continual learning and multimodal data fusion were identified as crucial elements that could provide accurate diagnosis and treatment recommendations.

**Impact:**

Our work is of methodological importance in that using artificial intelligence for molecular tumour boards is novel. We provide a consolidated proof-of-concept system that could support the end-to-end clinical decision-making process and positively and immediately impact patients.

**Conclusion:**

Augmenting a digital decision support system for molecular tumour boards with retrospective patient material is promising. This generates realistic and constructive material for human learning, and also digital data for continual learning by data-driven artificial intelligence approaches. The latter makes the future system adaptable to human bias, improving adequacy and decision quality over time and over tasks, while building and maintaining a digital log.

## 1 Introduction

The rapid growth in data volume in medicine, combined with technological advances in diagnostics and treatment options, is at least in theory allowing for healthcare to be tailored for every individual. Simultaneously, clinicians may face an overload of information, increasing the complexity of decision-making, and thereby the risk of non-optimal choices in complex human deliberations. Molecular tumour boards (MTBs) feature teams of experts from different specialties who meet to assess complex cancer cases to discuss individualised treatment strategies and to make recommendations according to the latest evidence. Principal members of an MTB for cancer are clinical oncologists/haematologists, pathologists, geneticists, bioinformaticians and molecular biologists, even though additional professional figures such as surgeons, bioethicists or oncology pharmacists could be included (1). In an MTB, decisions are supported by clinical guidelines, clinical studies, and the knowledge of the human participants in the meeting. Studies based on real experiences of MTBs advocate that recommendations from the multidisciplinary team improve the overall and progression-free survival of patients, compared to the decision of a single physician (2). MTBs appeared and gained significance within the emerging field of molecular oncology, especially with the development of next-generation sequencing (NGS) technology (3–5).

Precision medicine is an emerging field that aims to tailor medical treatment and prevention of adverse outcomes based on the individual needs of the patients, and it is decidedly interdisciplinary (6). While the traditional approach to clinical decision-making for medical treatment allows for generalisation and may benefit a population of individuals, the precision medicine approach wants to stratify populations and identify disease management that benefits all subgroups. It can be defined as *multimodal patient stratification and monitoring* as it helps separate patients into subgroups based on the clinical and molecular phenotypes, and also serves as a guide to how to best monitor each patient. The aim of precision medicine is to advance the development of targeted therapies, provide a more effective and personalised cancer journey, and achieve better outcomes (7). However, the large amount of molecular data and the increasing number of genomic alterations over time add complexity to interpreting them to give indications clinically (1). Apart from the morphological and histological features of the tumour, genetic alterations are now the target of personalised treatment, becoming molecular predictive biomarkers for agnostic drugs (2).

Lymphoma is a heterogeneous group of haematological malignancies that arise in the lymphatic system and where more than 80 different subtypes have been identified based on histopathology, immunophenotype, molecular genetics and clinical course. Classifying the lymphoma diagnosis correctly is critical to providing the right therapy, and efforts to sub-classify different lymphoma entities further into genetic subtypes have been developed by integrating NGS-based technologies (8) that facilitate a faster identification of genetic aberrations from tumour samples, at lower cost (9). Tumours should be classified based on genetic data to identify genetic aberrations (e.g., mutations, copy-number alterations, or fusions) (10), which would have higher clinical relevance (11).

MTBs play a prominent role in the clinical interpretation of these genetic variants. Specialists perform manual investigations into clinical studies and guidelines to interpret each variant and to understand their clinical utility (diagnostic, prognostic or therapeutic), if these are known. Specifically, clinical geneticists check knowledge bases for precision oncology such as Cosmic (12), My Cancer Genome (13) or Civic (14). In oncology/haematology, knowledge bases offer information about the relation between genetic variants and cancer type, as well as implications for diagnosis, prognosis, and treatment (15). BioLymph is an ongoing clinical prospective study at Karolinska Institutet and Karolinska University Hospital (16). It aims to improve knowledge regarding the impact of specific genetic aberrations on diagnostics and prognosis for patients with newly diagnosed lymphomas. For included patients, tumour samples are collected at diagnosis, and blood samples during treatment and follow-up. In addition, clinical data and patient questionnaires on comorbidity and quality of life, neuropathy and fatigue are collected at several time points. In BioLymph, discussion and interpretation of genetic results from the study are performed in MTB meetings in which the pathologist, clinical geneticist and the clinical oncologist/haematologists participate. The aim is that these discussions may help to identify high-risk patients and tailor treatments and follow-up strategies in the future. At the moment, BioLymph members are organising MTB sessions with retrospective patient data strictly for research purposes, with the aim of having MTBs soon integrated in the clinical routine.

The massive amount of potentially relevant health data increases complexity in the clinical interpretation and decision-making (17). Clinical decision support systems (CDSSs) are “computer systems designed to impact clinical decision making about individual patients at the point in time that these decisions are made” (18, p. 3.). These systems have gained importance in preventing medical errors and for improving patient safety. The three main parts of a knowledge-based CDSSs are the knowledge base, the inference (or reasoning) engine, and a user interface. The knowledge base usually employs IF-THEN production rules. The inference engine contains the formulas with rules and associations used to infer new knowledge or estimates from the input patient data. The output can be a list of possibilities, ordered by probability, or by urgency, such as which images a radiologist should look at first when the number is overwhelming. The major bottleneck is the maintenance of the knowledge base, as it needs to be updated as medical evidence and knowledge increase (18). Without knowledge bases that detail medical literature and expert knowledge, artificial neural networks and other machine learning approaches identify links and associations between, for example, symptoms and a diagnosis (19). Therefore, there is no need to hard-code rules and the strenuous maintenance of a knowledge base is avoided. While most of the developed models to accelerate data-driven health care are today based on unimodal data (such as genomic, radiological, or clinical data), multimodal AI methods could reshape precision oncology by integrating data cross multiple modalities by advancing patient stratification. Integrating modalities allows for the reduction of noise that one modality may cause and generates more accurate answers in predictions. This is an emerging field that still needs extensive research to discover the associations and underlying causal mechanisms at the molecular or cellular level, how to generate insights from new predictive models, or understand how to define data infrastructures that do not compromise patient integrity (20). Multimodal machine learning models have already been developed to stratify patients using multi-omics data such as genomic, proteomic, transcriptomic, and epigenomic data (21–23). One of the promising applications of multimodal datasets is CDSSs that learn policies from data and assist clinicians in their deliberations. This can produce systems with an increasing usefulness over longer time-scales, *via* continual learning (24) (25).

Many articles highlight the need for scalable informatics tools to support MTBs (1, 26, 27). Another essential need is data security and data privacy, to be able to ensure anonymity and secure sensitive data (26, 28). The challenges of clinical interpretation of genetic variants based on manual investigation of extensive literature may also be served by automated solutions (1, 28, 29). Because they differ in content and usability, specialists are forced to use multiple knowledge bases (29–31). Pishvaian et al. (32) developed a cloud-based virtual MTB, which integrated different data modalities such as the patient medical history, pathology results and -omics results to identify the most appropriate treatment option. It included an asynchronous chat and a rules engine that determine the recommended therapy. In Germany, an evidence-based decision system (cBioPortal (33, 34)) was developed, which guided the clinical interpretation of complex molecular data based on published guidelines (35). Other cancer variant interpretation tools have been implemented to unify multiple knowledge bases that mainly geneticists must manually check (31, 35–37). In addition, some efforts, including health informatics standards such as Fast Healthcare Interoperability Resources (FHIR) and openEHR have been implemented to enable data interoperability and integrate genomic information into the EHR (37, 38). At Karolinska Institutet, Tamborero et al. (39), under the umbrella of the Cancer Core Europe (CCE) network, developed an MTBPortal, which automatically unifies, interprets and reports -omics data analysis results from seven European cancer centres. The latter is defined as an academic CDSS, so it is not yet intended to be used in clinical routine. Regarding commercial solutions, tools like NAVIFY^®^ Mutation Profiles from Roche (40), QIAGEN Clinical Insight (QCI^®^) Interpret (41) or CureMatch^®^ Bionov™ claim to match NGS results to available therapies. We can also find NAVIFY^®^ Tumour Board, which is specifically designed to assist MTB meetings. This was evaluated through a prospective observational cohort study and demonstrated that digital solutions like that one could decrease the average time per patient case in discussions. However, the study results showed heterogeneity across cancer types (42). Even though commercial software offer nice front ends and end-to-end workflows, they have closed source proprietary architectures that prevent the integration with other systems and knowledge bases that may be more relevant for the tumour or cancer type (31). AI-based decision support could be integrated into MTB meetings to support treatment choice and overcome the limits of human cognitive capacity. An early solution was the IBM Watson for Oncology, an AI-based CDSS for lung, breast, and prostate cancer. Evaluation articles report on the concordance between recommendations made and MTBs (43–45). The concordance was very good but it varied with the stage of the cancer type. It is manifest that discordance may be due to the difference between countries on national guidelines and recommendations, ethnicity facts or insurance coverage compliance (43). The “Tumor Profiler Study” (46) is an observational trial which aims to support the clinical decision-making with NGS, similar to the BioLymph study.

In addition to their use for precision oncology, the implementation of MTBs may have drawbacks. For example, their wide implementation will affect the more specialised conferences already in use at clinics, the relation to which would need to be investigated. Literature revealed few attempts to develop digital health solutions for supporting clinicians, and most of them are for research purposes. Most of the solutions use the traditional approach by developing knowledge-based and rule-based systems. Even though these systems are transparent and evidence-based, they require great effort to maintain and update continuously, as knowledge increases. Few studies have investigated how to support the end-to-end planning and decision-making process considering the latest advances in AI and involving end-users. Our research objectives were thus to identify the specific requirements for a CDSS for MTBs at Karolinska University Hospital; to explore the inclusion of AI for multimodal data analysis into an MTB tool; to create a first prototype of the user interface based on the requirements, and to formatively evaluate the results with cancer care experts involved in MTBs.

## 2 Methodology

This study used the Design Science Research Methodology (DSRM) (47) for the design and evaluation of the proposed artefact. We followed Peffers et al. (48), who developed a consensus-building process model based of activities in a nominal sequence while possible to iterate and repeat to improve the final prototype. Even though DSRM supports both quantitative and qualitative research methods, our study is purely qualitative.

The DSRM consists of six activities in a nominal sequence but it is possible to iterate and repeat to improve the result. The activities are: (i) Problem Identification and Motivation, (ii) Define the Objectives of a Solution, (iii) Design and Development, (iv) Demonstration, (v) Evaluation, and (vi) Communication. In our research, the stakeholders were aware of the problem, which helped defining it and hypothesizing that a custom artefact could be proposed to support MTBs (i). A requirements elicitation and analysis was then performed to define how the proposed artefact would address the problem (ii), using data from the environment (47). The requirement analysis was approached by first performing a scoping review to identify general requirements of similar solutions and then by gathering needs and requirements from principal stakeholders. An artefact design was proposed by creating a requirements specification based on the list of requirements collected (iii). To demonstrate the use of the artefact, scenarios were considered (iv). Domain experts evaluated the proposed artefact in a second round of interviews by validating the requirements through the mock-up (v). The results of the process were then described in the present publication and disseminated to the precision medicine task force at Karolinska University Hospital and Karolinska Institutet.

### 2.1 Scoping review

The scoping review was based on scientific literature from 2017 to 2022—since the field and technologies are both accelerating fast—for the PubMed, Web of Science and IEEE Xplore databases. In addition, the review identified relevant research following the snowballing technique and by hand-searching key sites. An article to be relevant should include at least one eligibility criterion:

- Data-related challenges on current MTB meetings
- Implementation or potential development of tools or systems supporting MTBs
- Development or integration of AI-based models intended to be used in MTBs
- Development of multimodal data integration strategies intended to be used in MTBs
- Design and implementation of multimodal data-based systems
- The above criteria must be particularly based on -omics data as well as on other relevant data modalities

Articles in another language than English, unavailable full text, or a non-relevant title or abstract were excluded (see **Supplementary Materials** for PRISMA chart and further details). A combination of controlled (MeSH Terms) and non-controlled vocabulary was used in the search queries, and multiple searches were used in the different databases (**Table 1**). A first broad search included “precision oncology” OR “precision medicine” to identify data-related challenges on MTB discussions. Other searches included “artificial intelligence” and related terms to identify AI-based models and “multimodal” or “multi-modal”. Since IEEE Xplore is a database for technical literature in engineering and technology, the search strategy focused on finding health information systems that integrate multimodal data and examples of big data infrastructures in precision medicine and clinical settings.

**Table 1:** Search strategy for scoping review.

Database	Search strategy
PubMed	[(tumor board) or (tumour board)] and [(precision medicine[MeSH]) or (precision oncology)]
PubMed	[(tumor board) or (tumour board)] and [(artificial intelligence[MeSH]) or (machine learning) or (deep learning)]
PubMed	[(tumor board) or (tumour board) and [(multimodal) or (multi-modal)] and [(integration) or (data)]
Web of Science	[(tumor board) or (tumour board)] and [(precision medicine) or (precision oncology)] and (system)
Web of Science	[(tumor board) or (tumour board)] and [(artificial intelligence) or (machine learning) or (deep learning)]
IEEE	[(multimodal) or (multi-modal)] and (precision medicine)
IEEE	(big data) and (precision medicine) and (integration) and [(system) or (platform)]

### 2.2 Semi-structured interviews

The results from the scoping review were used to prepare the interview guide (see **Supplementary Materials**) for the semi-structured interviews. The chosen method to analyse the interview was thematic analysis (49), for identifying patterns or themes within data and group them according to similarities (while content analysis should aim to evaluate patterns to determine their frequency or relationships) (50). The initial code production started by identifying interesting sections of the text and assigning labels to them. Codes were meant not to be redundant and also interchangeable. When all data had been coded, we sorted the codes into themes by following a hybrid approach. Initially, some themes were generated deductively from prior research. New themes were then identified inductively from the data, regardless of the relation to the questions asked. Because some themes could belong to a higher level in this categorization, subtheme groupings generated the main themes. Themes, subthemes, and codes were then validated in an iterative process. Finally, all entities were renamed, as necessary. We followed trustworthiness criteria of credibility, transferability, dependability, and confirmability (51). System requirements can be classified as functional or non-functional requirements. The former describe the services of the system, how the system should react to particular inputs, and how it should behave. The latter are constraints on these services or functionalities and may apply to the entire system, rather than to particular characteristics (52). Therefore, the requirements specification was written based on the results of previous methods, the scoping review and one-on-one interviews, following the Volere Requirements Specification Template (53).

1. Project drivers: purpose of the project, stakeholders, and the intended users of the product.
2. Project constraints: the limitations and the restrictions on the project, vocabulary of the project and relevant facts and assumptions.
3. Functional requirements: the functionality of the product with the scope of the work and product and the functional and data requirements. This section is complemented by use case diagrams, which model the interactions between the users and the system and identify the boundaries.
4. Non-functional requirements: the qualities of the product such as look and feel, performance, security or legal requirements.
5. Project issues: problems relevant to the project that builds the product. The last point in this section is “Ideas for Solutions”, and is meant to be the place to write the ideas about potential solutions not included in the real requirements.

### 2.3 Evaluation

Prototyping is one of few tools that can help users to develop a real sense about final systems that are not yet implemented. In addition, users may find new ideas for requirements, strengths and weaknesses. With this intention, a digital low-fidelity mock-up was designed for the evaluation, based on a scenario (54) for a complex lymphoma case, as follows.

**Initial assumption:** Maria is a haematologist who has identified a lymphoma patient with a complex diffuse large B-cell lymphoma and needs to schedule an MTB to confirm the diagnosis, elaborate on tumour characteristics and discuss the best treatment. She had four additional complex patients, so she needs to plan and coordinate an MTB meeting for five cases.

**Normal:** She logs in to the system and starts the preparation of the new tumour board. She writes a brief description, the meeting date, time, duration, location, the other expert participants and the list of patients. She saves the information and opens the board on a new page. She can view a few details of the five patients and can click on them to extend the information. She opens the first diffuse large B-cell lymphoma patient profile and can choose between ‘Health Record’, ‘Clinical Genetics’, ‘Pathology’, and ‘MTB Report’. She goes through all the tabs to check that all the information has been integrated well and explore the clinical context of the patient and the pathological data including the immunohistochemical results and the gene mutations. She can use a feature that allows her to retrieve gene variant or mutation information from PubMed publications. Then, Maria explores another feature where she can see a list of diagnosis and treatment recommendations that AI models have predicted with levels of confidence and list of the features that contribute the most to that decisions. Maria has now prepared the board and has checked all the information of the patient. The other expert participants also have access to the system and can check the patient data and add information accordingly. During the meeting in a few days, Maria will repeat the steps from the profile of the patient, together with the other attendees, who have also access to the system. All the information and features will support the decision-making process. Finally, she can download the file on her device and export it to the patient medical record. The MTB can follow with the next patient.

**What can go wrong:** The patient information has not been integrated. Maria and the other specialists should add the data manually.

This corresponds to a single scenario and it does not consider all possible risks, such as over- or under-diagnoses if data is not interpreted correctly. There is also a risk not to benefit from the advantages of the software when following manual processes, such as poor patient- and disease stratification, or to apply treatments with little or no benefit.

For the second round of interviews, the chosen scenario constrained our choice to clinical physicians with experience of organising MTB meetings. Two of the five interviewees met this criterion. Additionally, clinicians that participated in MTB meetings at the hospital but did not participate in our study were invited, to reduce bias. At the beginning of the interview, the interviewer gave a brief description of the experiment, main findings of the study so far (key challenges and key user needs), and the characteristics of the potential solution. The introduction followed by the description of the scenario and an explanation on how they can interact with the mock-up and its limitations. Then, the interviewer shared the link of the digital mock-up and asked the participants to share the screen. Participants were asked to complete some tasks, according to the scenario, while they were encouraged to express their sentiment through the user interface. Since the mock-up was not fully interactive, the interviewer guided the participant through the scenario and interface. After the tasks, they were asked the following questions: (i) Would this system satisfy your needs?, (ii) Do you miss any feature or functionality that may help?, and (iii) What do you think could be improved?.

### 2.4 Ethical considerations

This study considered the protection of dignity, integrity, confidentiality, right to self-determination, and privacy of personal information (55). The methodology was designed to minimise the risk of intrusion into the autonomy of the interviewees. All the participants were contacted *via* email and once the invitation was accepted, an informed consent form was sent, to be signed prior to the interview (**Supplementary Materials**). Therefore, the interviewees were aware of how their data would be used.

## 3 Results

During the scoping review, articles that included insights that could serve as initial requirements of a CDSS for MTB meetings using -omics and other data modalities were annotated and broadly categorised (see **Supplementary Materials**). The results of the interviews are described partially based on a consolidated criterion for reporting qualitative research (56), which consists of (i) research team and reflexivity, (ii) study design, and (iii) data analysis and reporting. Only the items that corresponded to post-interviews information are included here. Since the sampling method was purposive, only professionals who could provide rich and relevant information were interviewed (see **Supplementary Materials**). The thematic analysis resulted in seven main themes, 26 subthemes, and 130 codes (see **Supplementary Materials**).

**Figure 1:**
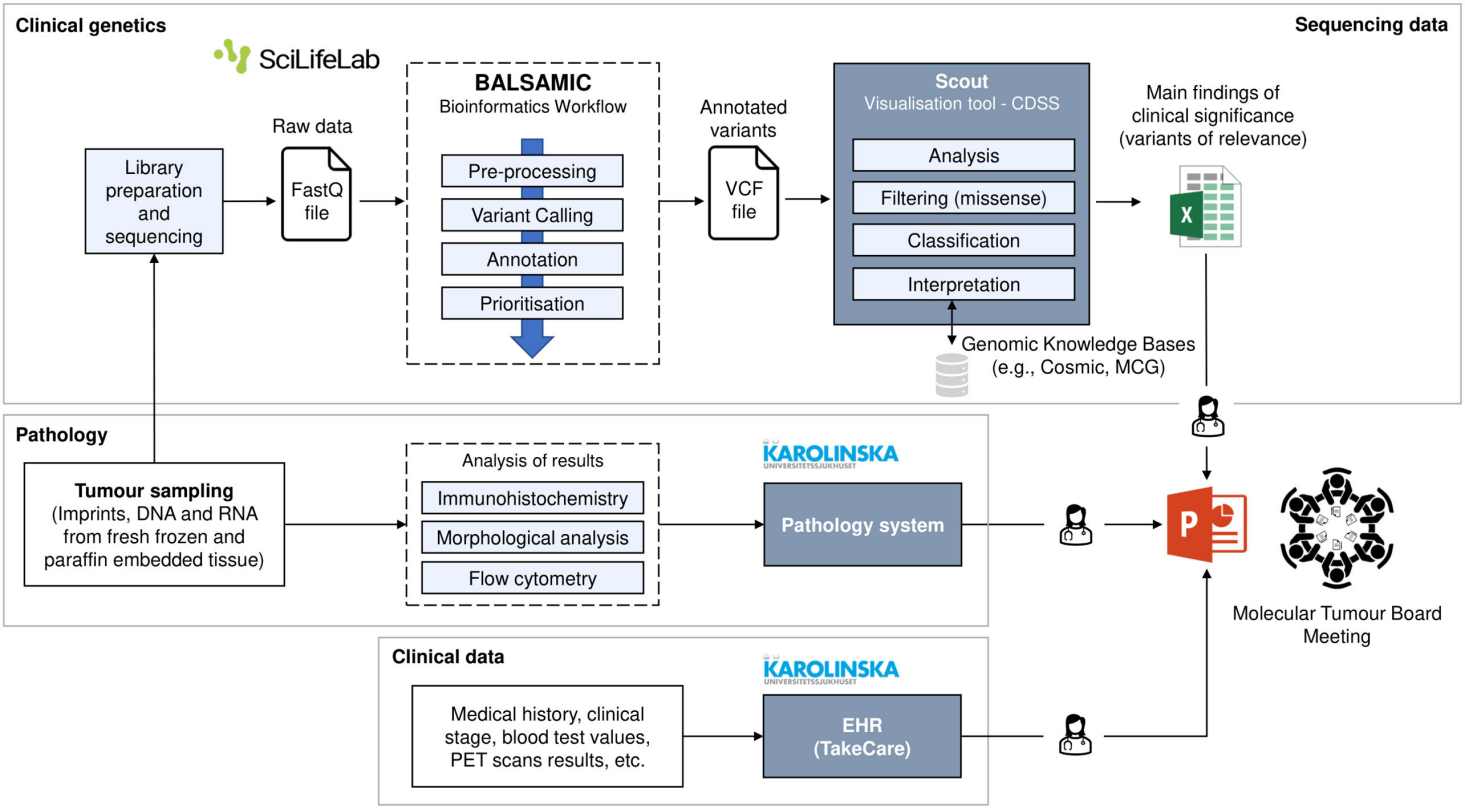
Current workflow for MTB meetings in the BioLymph study.

*Theme 1: MTBs for Lymphomas.* vance of the MTBs and their meetings. As mentioned in the Introduction chapter, the meetings are not currently taking place because new technologies and methods that provide the genetic data (NGS-based technology) are not established in the clinic yet. Instead, they use retrospective patient data for research purposes on an experimental level. However, the goal is to establish the meetings in the clinical routine during the diagnostic work-up. The diagnosis period lasts approximately two weeks, and the data collection and analysis now take more than that. Nevertheless, other professionals in the hospital can attend the meeting, such as bioinformaticians, to support the evaluation of results from a data point of view or other physicians and clinicians involved with the lymphoma patient case. Therefore, it is a significant multidisciplinary group. All the participants agreed that MTB conferences are an essential meeting point and a decision-making process. One participant said that allowing the discussion of molecular data helps, for example, to refine the lymphoma subtype diagnosis. The oncologist pointed out that it is also an educational opportunity where the discussion of one patient may help other patients. The meetings are prepared from the clinical side, where one clinician assumes the coordinator role. The coordinator sends the patient identifier to the other specialists, who should collect the corresponding data of the patient. So, the geneticists would collect the results of genetic data analysis, pathologists would collect pathological data, and the clinician would collect the clinical data about the patient, which would provide the essential clinical context. Data is collected and sent manually to the coordinator, who prepares a slide presentation, in which all the patient data, results, and findings are presented, and acts as the supporting tool to run and guide the meetings (**Figure 1**). DNA and RNA extracted from tumour samples are converted to sequencing libraries. This raw data is delivered in FastQ files. Sequencing data is analysed through BALSAMIC (57), a bioinformatics analysis pipeline for somatic mutations in cancer. In short, the process consists of identifying gene variants from sequence data (“Variant Calling” in **Figure 1**), assign information to gene variants (“Annotation”), and give a prioritisation score to annotated variants (“Prioritisation”). Then, the prioritised variants are uploaded in a VCF (Variant Call Format) file to Scout, a custom-developed decision support system at the SciLifeLab Clinical Genomics facility at Stockholm. Clinical geneticists use Scout both as an analysis tool and a local database, which helps remove artefacts, such as locally recurring variants at low frequencies. Scout provides them with links to the international databases for cancer genetics, in which they then search for the specific variant that they are interested in. The databases are regularly updated and curated so that the data there is relevant and can be trusted, since the information builds on peer-reviewed results from PubMed. They prefer dynamic links rather than having all the data extracted from Scout because that would require version management and constant checks for updates. They use a local database (Scout and Alamut), Gnomad, Cosmic, My Cancer Genome, oncoKB, ProteinPaint (St.Jude cloud), and VarSome. The latter provides them with good predictions for the variants not previously published.

*Theme 2: Relevant Information for Discussion.* White boxes in **Figure 1** represent the information that they are using today. However, since BioLymph is a prospective ongoing project, they are working on generating other types of data to introduce in the discussions. This theme is based on the relevant information from each speciality that needs to be presented in an MTB meeting for lymphomas and user requirements (UR).

- UR01 - As a user, I need to have the complete clinical context of the patient to enable decision-making.
- UR02 - As a user, I need the following clinical data to support my decisions:
  - Patient demographics: age, sex.
  - Comorbidities: number and type of comorbidities.
  - Laboratory test results: blood test results (they will show the status of lymphoma and other organ functions), lactase dehydrogenase (LDH) test as a prognostic factor, tests of bone marrow, kidney and liver functions, biopsy.
  - Imaging studies: CT or PET CT scans results, which will show the stage of the disease, spread of disease and location (one or multiple).
  -Medication: treatment received, treatment response.
  - The current status of the patient.

Sequencing data is the core information in MTB discussions, helping to confirm the diagnosis, but also to evaluate tumour characteristics carrying prognostic and/or predictive potential, and how to choose the right treatment. There are genetic alterations or variants that are well-known, making it easy to interpret both the diagnosis and prognosis. However, uncommon alterations, rare or new, spur the clinical geneticists to search knowledge bases to check their meaning. In those cases, the relevance of the MTB increases since the findings must be discussed with the other clinical and pathological data.

- UR03 - As a user, I need the following genetic data to support my decisions:
  - Novel and actionable genetic variants.
  - Somatic mutations.
  - Translocations for diagnostic and prognostic.
- UR04 - As a user, I need the following pathological results to support my decisions:
  - Lymph node biopsy.
  - Immunohistochemical stainings.
  - Morphology of the cells and their interaction.
  - Flow cytometry.

Metadata is very important for reproducibility and trace decisions, as it can add an extra layer of information in the decision-making and reduce false positives.

- UR05 - As a user, I need to know metadata from the following elements and processes:
  - Experimental, sample, and biomaterial metadata.
  - Metadata from sample collection, library preparation, data processing, and software versions.

*Theme 3: Involved Tools and Other Related Solutions.* The current supporting tools are Scout (Clinical Geneticists), PowerPoint, and occasionally, Cisco, for external communication to other hospitals to discuss complex cases. When participants were asked if they knew any system that could support their decision-making process, they mentioned the commercial solution from Roche (NAVIFY) and the MTB Portal by Tamborero et al. (39). They stated that in order to ensure patient integrity, those tools cannot be used because they would have to send sensitive NGS-data to external servers. Moreover, the portal currently does not support haematological malignancies.

*Theme 4: Problems and Challenges.* Data is stored in different information systems, which only the specialists of each type have access to. Moreover, in order to prepare the meeting and interpret the results, they have to look into different knowledge bases (e.g., Cosmic, MCG, OncoKB), since not all of them are continuously updated, and new variants are discovered all the time. As they check PubMed, they may find information not yet registered in the cancer genetics knowledge bases. This represents a high workload on geneticists and oncologists in particular, and clinicians stated several challenges (CH) along the interviews:

- CH01 - It is challenging to collect, organise, and summarise all the data for a single patient case and make sure that the knowledge reaches the patients.
- CH02 - Genetic data is very difficult to interpret and to make it easily available and presentable in clinical practice.
- CH03 - Particularly for lymphoma, it is also difficult to stratify patients into all the categories at the clinic.
- CH04 - It is a challenge to make the work process more rapid and smooth for the patient to benefit.
- CH05 - Moreover, they are aware that the genetic landscape will become even more complex and it is crucial to know how to distil the most important features from these complex datasets.

*Theme 5: User Needs.* Interviewees highlighted the need of having user-friendly layouts, asked for an interactive panel or dashboard, and stressed the data quality aspect. e treatment selection and treatment stratification based on genetic data. Moreover, the outcome of the meeting should be reported somehow.

- UR06 - As a user, I want the system to be integrated in the hospital, rather than an external system.
- UR07 - I would like to have an automatic retrieval of patient information.
- UR08 - I want to be able to input the data and see the data from other specialists prior to the meeting date.
- UR09 - I want a system that support collaboration within the participants.
- UR10 - I need a system easy to use, self-explanatory and that presents the information in a manageable and nicely presented way.
- UR11 - I need the most updated information all the time for the accurate decision-making.
- UR12 - I need help in the interpretation of the status of the mutations, and know the relation at diagnosis, prognosis, and predictive level.
- UR13 - I need to document in a final report who participate, when, what has been discussed, and the decisions.
- UR14 - I must ensure that we have an appropriate level of protection for patient data privacy.
- UR15 - I would like to have predictions based on the integration of genetic data and the other data modalities.

*Theme 6: Technical Considerations.* The first issue that the data expert pointed out was the difference between building software in an academic environment and a clinical one. The clinical context is more complex since it requires very well-planned flows and stricter legal agreements. In addition, the system would use translational research data which is difficult to integrate in the clinical routine. Systems that integrate large datasets and have complex computational pipelines need certain types of storage and resources. The IT specialist explained the differences between local processing and cloud computing. While local processing has limited storage capacity, cloud resources do not. However, they are expensive and require legal agreements for sensitive data management. On the other hand, local processing may be beneficial if there is the right expertise and the further maintenance is ensured. Both types, as well as hybrid solutions are valid, if the legal considerations are well-covered. According to the IT specialist, a system to support MTBs in the clinical routine would reduce the pressure on clinicians, support efficient processes, and reduce errors.

*Theme 7: Artificial Intelligence.* Even though a common view amongst interviewees was that AI is a black box, the majority agreed that AI could give the next step in MTB meetings. Some of the potentials that they viewed were the reduction of false positives and false negatives and that it could narrow the gap between over-reporting and under-reporting. Clinicians stated that AI could give the risk, prognosis and the right treatment based on the genetic aberrations that the geneticists identify. The participants on the whole demonstrated a positive attitude towards AI and knew it is a key factor to distil the complex data that they need to manage.

### 3.1 Stakeholder analysis

**Figure 2:**
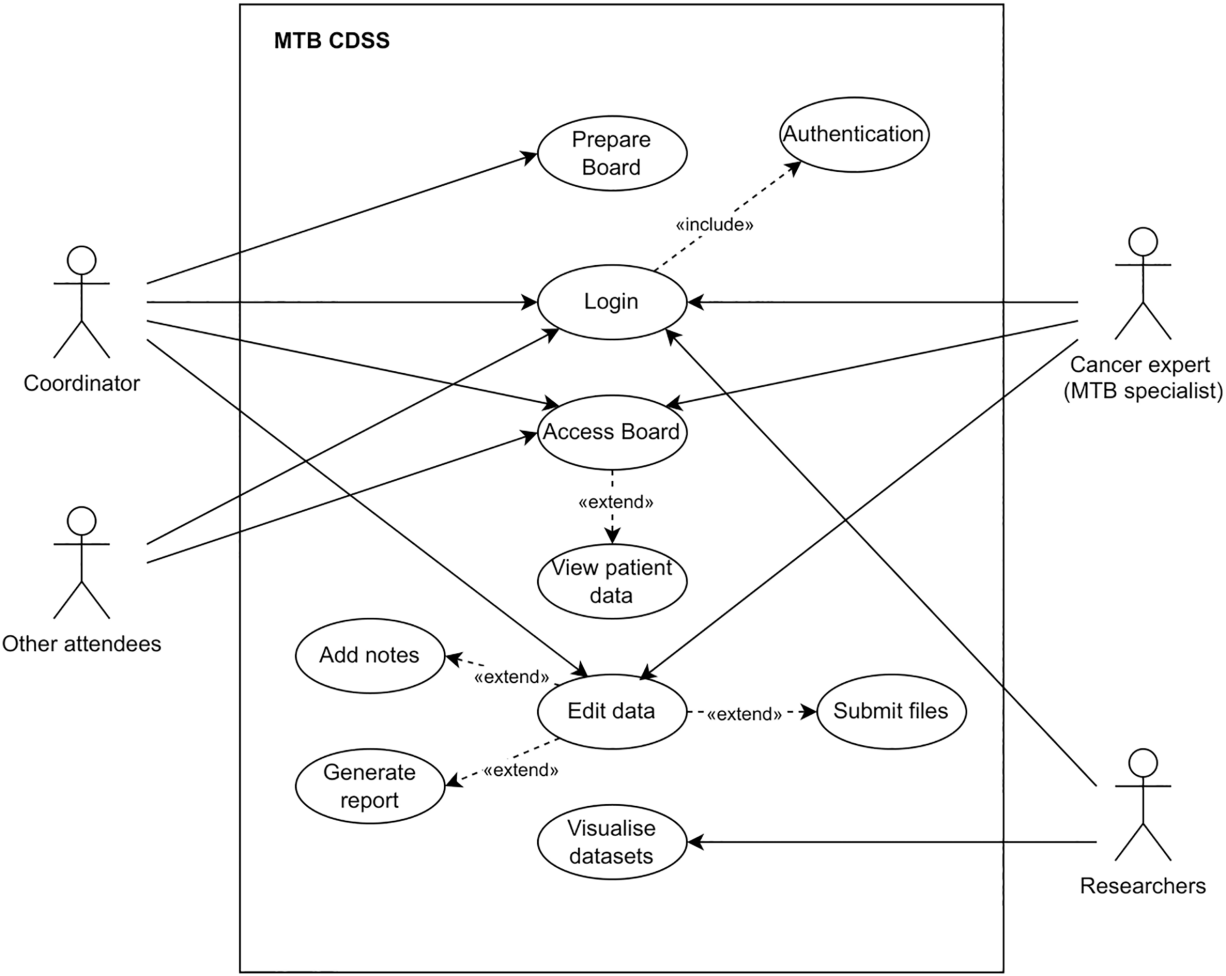
UML Use Case Diagram for the CDSS.

The user requirements (URxx) identified represent the high-level abstract requirements of the system. User requirement statements were then translated to system requirements (SRxx), which provide more detailed descriptions of what is to be implemented. Some additional requirements from the literature review (LRxx) aligned with the user needs were also included to the list of system requirements. Project drivers are people with an interest in or can influence the project, as well as the intended end-users (**Table 2**). The degree of involvement is divided into *low* if stakeholders are only recipients of knowledge, *medium* if stakeholders are asked to provide their knowledge and, *high* if stakeholders are collaborative actors in which their knowledge helps to shape the research process (59). A use case diagram determines the system boundaries between the users or actors and the system that is about to build. Four actors participate in the use cases within the scope of the artefact, and they are located outside the system boundary (the rectangle in **Figure 2**).

- A cancer expert or MTB specialist is the person who uses the system to check and upload patient data according to their speciality.
- A coordinator is the person in the hospital who uses the system to prepare an MTB and have write access in its totality. The coordinator is also an MTB specialist.
- An attendee is any person involved in the patient journey who uses the system with read-only access.
- A researcher is the person who uses the system to access the different databases de-identified.

**Table 2:** Stakeholder analysis of the project based on the Volere Stakeholder Analysis Template (58).

Role	Rationale	Involvement
MTB specialists	Interact with the system (end-users).	High
MTB attendees	Access the system with limited privileges (end-users).	Medium
Karolinska University Hospital	Benefit from the system in terms of status and influence.	High
Patients	Benefit from the output of the system.	Low
SciLifeLab	Collaborate in the design and have interest in the output.	Medium
Karolinska Institutet	Collaborate in the design and have interest in the output.	Medium
Researchers	Access the system with limited privileges and contribute to the development.	Medium
Legal experts	Support development according to laws and regulations.	Medium
System designers	Design and support development of the system.	High
Software developers	Build the system.	High

The set of use cases that represent single interactions are explained below. A use case can extend one or more use cases as optional additional behaviours, or can include a mandatory use case, as part of the base use case.

- The use case **Login** provides all the actors with the right to access the system. It includes the behaviour of another use case, **Authentication**, and thus, Login depend on the execution of Authentication. Then, the actor must be authenticated in order to login on the system.
- The use case **Prepare board** provides the Coordinator to input administrative details about the tumour board such as who is going to attend or which patients are going to be discussed.
- The use case **Access Board** provides the coordinator, cancer experts, and other attendees to access the tumour board in the system. The extended use case **View Patient Data** provides the actors to access all the information about the patient.
- The use case **Edit data** provides the coordinator and cancer experts to modify information in the view of the patient. The extended use cases **Submit files**, **Add notes**, and **Generate report** provide the actors to upload new files that could not have been integrated, introduce what is discussed in the meeting or final recommendations, and save the final report.
- The use case **Visualise datasets** provide researchers to view the processed datasets.

### 3.2 Functional and data requirements

- SR01 - The system shall display the clinical context of the patient from the EHR: chief compliant, history of present illness, past medical history, laboratory test results, and diagnostic test results. (UR01, UR02)
- SR02 - The system shall display the results of patient questionnaires (PROMs). These shall be stored within the EHR system. (LR08)
- SR03 - The system shall display or allow the upload of the genetic variants. (UR03)
- SR04 - The system shall display the list of gene mutations in relation to the diagnosis, prognosis, and predictive level. (UR12)
- SR05 - The system shall display the pathology results from the pathology system. (UR04)
- SR06 - The system shall display the metadata from the bioinformatics pipeline and analysis of sequencing data (UR05).
- SR07 - The system shall display the latest information. (UR11)
- SR08 - The system shall allow the manually upload from additional systems that cannot be integrated automatically.
- SR09 - The system shall allow to review or add information before the meeting date. (UR08)
- SR10 - The system shall allow users to view and edit the patient profile at the same time to facilitate the discussion of tumour board cases. (UR09, LR18)
- SR11 - The system shall allow the generation of a report by writing in free text fields editable by all the participants with write access. (UR13, LR19)
- SR12 - The system shall display the results of advanced predictive models based on multimodal data. (UR15)
- SR13 - The system shall predict a list of treatment recommendations based on the multimodal data integration. (UR15, LR23)
- SR14 - The system shall allow the interactions with the database through a portal. (LR38)
- SR15 - The system shall retrieve patient data automatically from the integration with HISs. (UR06, UR07)
- SR16 - Data from other systems shall be standardised to enable automatic integration processes. (LR02, LR03)
- SR17 - Data shall be stored in interoperable and interrogatable databases. (LR04)
- SR18 - Data warehouse shall store raw data and metadata. (LR10)
- SR19 - Data warehouse shall integrate data from different types or modalities. (LR11, UR15)
- SR20 - Data warehouse shall support HL7/FHIR as healthcare informatics standards and the connection to statistical tools such as R and Python. (LR14)
- SR21 - Data warehouse shall support the connection between data and AI frameworks such as Tensorflow, Keras, or PyTorch. (LR15)
- SR22 - The system shall enable real-time data collection. (LR07)
- SR23 - The system shall allow different users to log into their account and access the tumour board. (LR20)

### 3.3 Non-functional requirements

#### 3.3.1 Usability requirements

- SR24 - Data shall be presented in a user-friendly format before the time of the MTB meeting. (UR08, LR16).
- SR25 - The system shall have a user-friendly and intuitive UI. (UR10)
- SR26 - The system shall have a modern user UI tailored to the needs of the clinical initiative. (LR37)
- SR27 - The UI design shall follow usability principles (e.g., General Usability Heuristics from Jakob Nielsen). (LR40)

#### 3.3.2 Security requirements

- SR30 - The system shall have a security framework to restrict authorisation and set authentication schemes to allow access only with valid credentials. (LR36)
- SR31 - The system shall have a secure and robust data warehouse to ensure patient data privacy. (UR14, LR34)
- SR32 - Computational pipeline shall be stable, robust, reproducible, and traceable. (LR26)
- SR33 - Machine learning models shall not be black boxes and must consider explainability approaches. (LR27)
- SR34 - The system shall follow the FAIR (Findability, Accessibility, Interoperability, and Reusability) guiding principles for scientific data. (LR28)
- SR35 - The system shall implement an audit trial of the data the decisions were based on. (LR29)
- SR36 - The system shall ensure data quality. (LR30)
- SR37 - The system shall be transparent. (LR31)
- SR38 - The system shall be scalable. (LR32)

#### 3.3.3 Performance requirements

- SR28 - The system shall allow an efficient data analysis workflow. (LR09)
- SR29 - The UI design shall enhance the usability in an efficient and effective way. (LR39)

#### 3.3.4 Legal requirements

- SR39 - The system shall follow the EU Regulations for the analysis of personal data: the GDPR, the 2017/745 Medical Device Regulation (MDR), and the recommendations of the European Data Protection Board (EDPB) under *Schrems II* (60).
- SR40 - The system shall follow specific EU Regulations for AI-based systems, such as the EU Regulatory Framework Proposal on AI (Artificial Intelligence Act) (61).
- SR41 - The system shall follow the new regulation of the European Commission, the European Health Data Space (EHDS), which supports the use of health data for better healthcare delivery and research, among others (62).
- SR42 - The system shall follow the IEC 62304:2006 Medical device software - Software life cycle processes.
- SR43 - The system shall follow the ISO 25720:2009 Health informatics - Genomic Sequence Variation Markup Language (GSVML) for genomic data exchange.

### 3.4 Solution design

**Figure 3:**
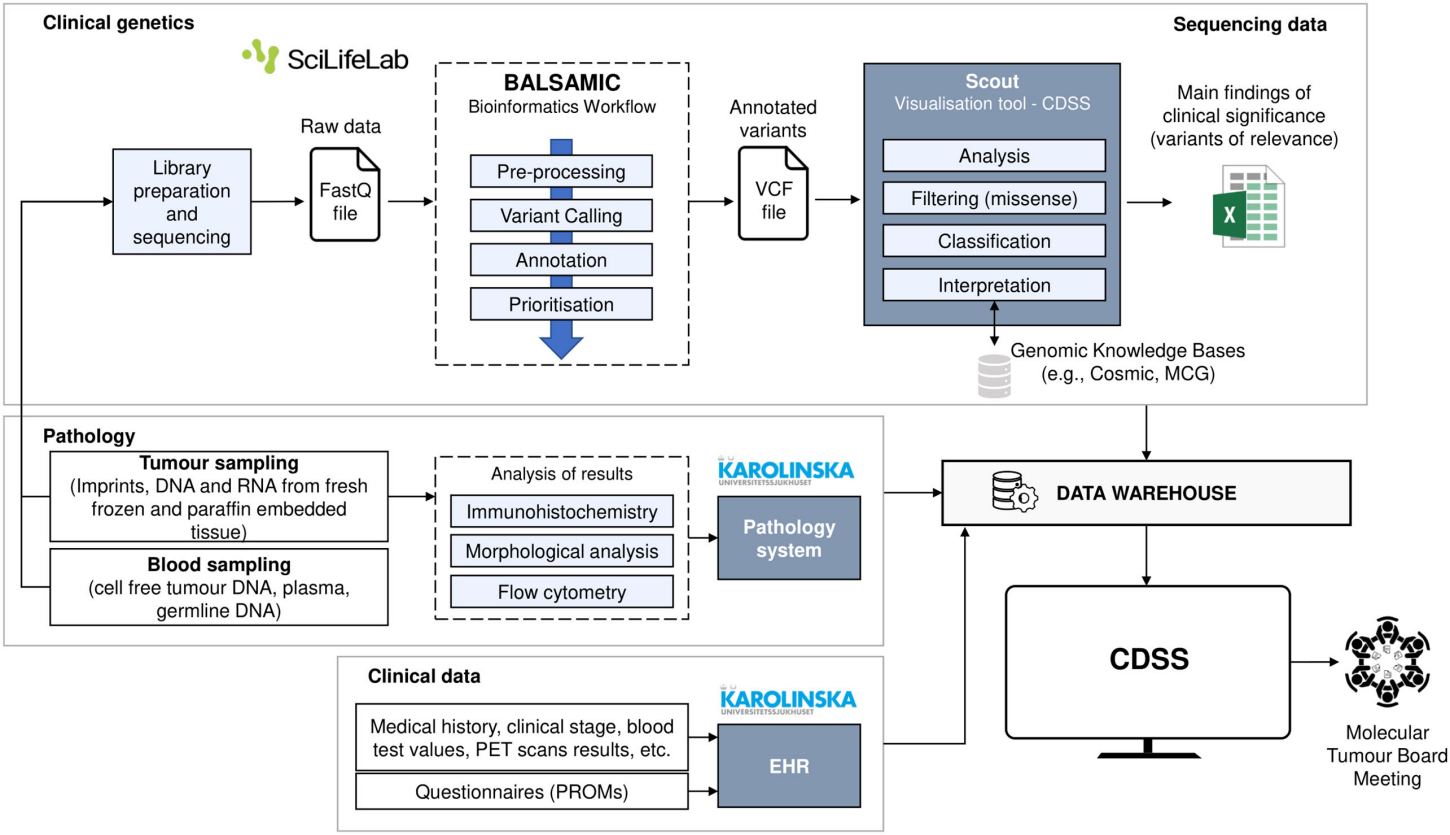
Future workflow for MTB meetings in the BioLymph study.

A Web-based information system could provide the opportunity to login with valid credentials, access all relevant patient information prior to the meeting, check the clinical evidence of genetic variants in PubMed, check diagnosis and treatment recommendations based on multimodal data fusion and AI, and report the outcomes of the discussion (**Figure 3**). Comparing to the current workflow (**Figure 1**), more data has been considered, as the BioLymph study aims to analyse such data points in the near future. Moreover, information would be collected and integrated automatically in a central repository (data warehouse), instead of collecting it manually, and results presented in slides format. We faced the challenge (CH01) to gather all the patient information from the EHR, PROMs, genetic results or pathology reports and organise them. In addition, the meeting had to be planned and coordinated efficiently, with real-time data integration with the other services. Alternatively, the system would have an option to upload files, so that files with relevant variants could be presented. The preparation of the patient case is a time-consuming process (CH04). There is no tool that covers all knowledge bases required to clinically interpret all molecular variants (CH02) but options could be:

- The system accesses the information for cancer variants from knowledge bases through a public application programming interface (API). The API needs to be provided with access options for other software. However, the integration may be error prone and complex due to differences in programmatic interfaces, data models or formats (31). Efforts to ensure interoperability and robust algorithms would be needed.
- Data from PubMed can also be integrated through eUtils (Entrez Programming Utilities), which provide an interface into the Entrez query and database system at the National Center for Biotechnology Information (NCBI). The Entrez system consists of a set of databases on biomedical data, including gene records and biomedical literature (63). Among the NCBI tools, there is LitVar, a semantic literature search engine for genomic variants (64). It allows for the retrieval of variant related information and its relation to close entities such as genes, diseases, and drugs. LitVar provides an API to enable the access to the results.
- Since the geneticists are already using Scout for visualising and interpreting gene alterations with direct access links to those cancer knowledge bases, the system could support the upload of the resulting CSV file. Then, the system can integrate the PubMed tool, LitVar, to check the latest evidence. This was our selected option.

Another relevant user goal was to improve patient stratification (CH03). Users would highly appreciate, for example, a solution that could aid the prediction of treatment response and classification of high-risk lymphoma patients, defined as treatment refractory disease or rapid relapse within one or two years (depending on lymphoma subtype), based on available biomarker data and other relevant patient information. Large and different data sources require the need of advanced analytical approaches and a robust data warehouse. The proposal includes a recommender system based on continual learning. It gives diagnosis and treatment recommendations for single patients based on multimodal data fusion and AI. The last challenge (CH05), making decisions based on large amounts of data and distill the most relevant features will become more complex. The idea is to provide MTBs with a list of predicted diagnoses and treatments, sorted by level of confidence of the AI models. Users will report the consensus decision in the system, and the new patient data and resulting outcomes will feed and update the model. Therefore, the model is progressively learning and improving its performance. In addition, to comply with security requirements about transparency (SR37), the interface will display an explanation about which features contribute to the recommendations. Lastly, users will write what has been discussed during the meetings and the final consensus in the system. This will generate a PDF file that could be downloaded or sent directly to the EHR. At the meta-level, such files can also be used as MTB meeting logs, for continual machine learning.

**Figure 4:**
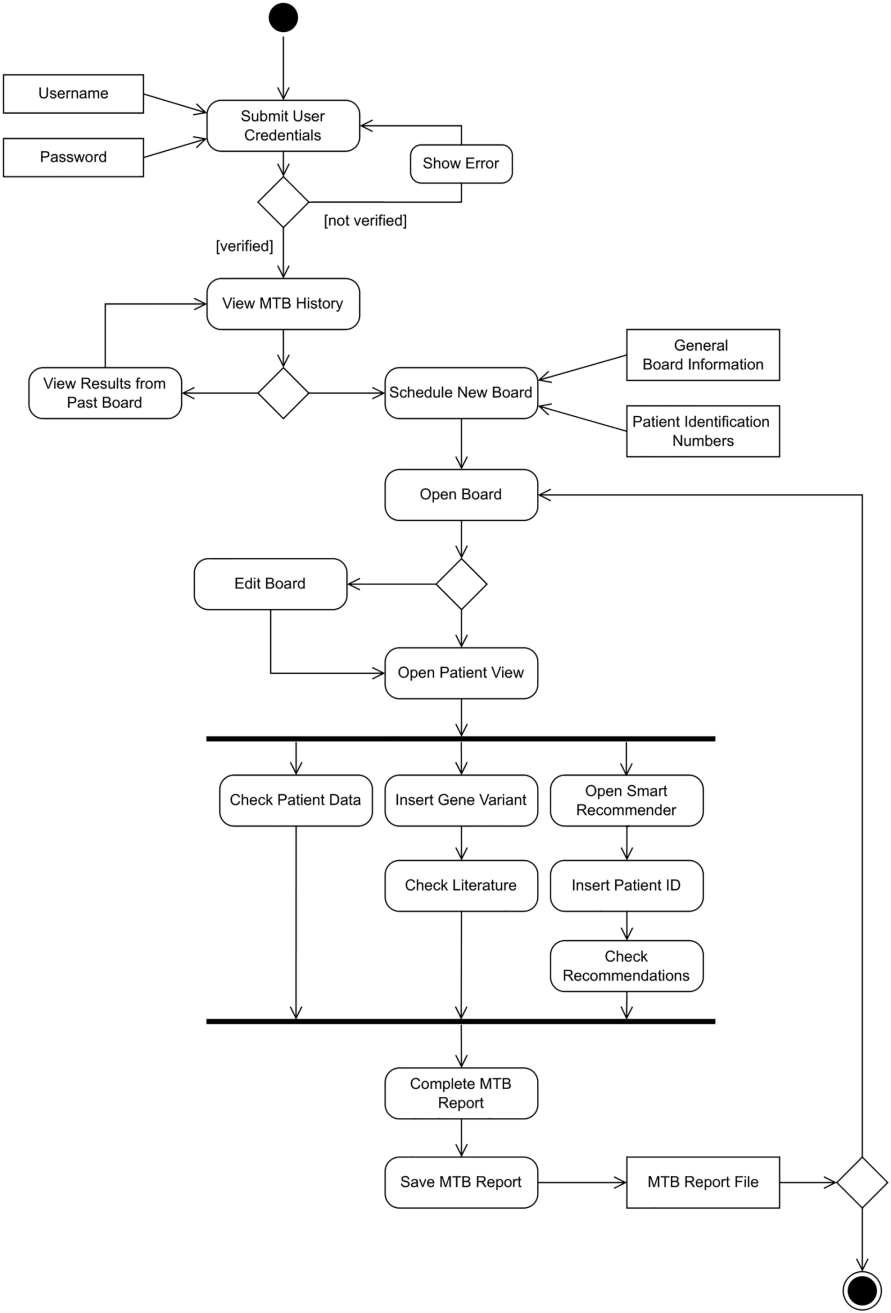
UML Activity Diagram for the CDSS.

**Figure 5:**
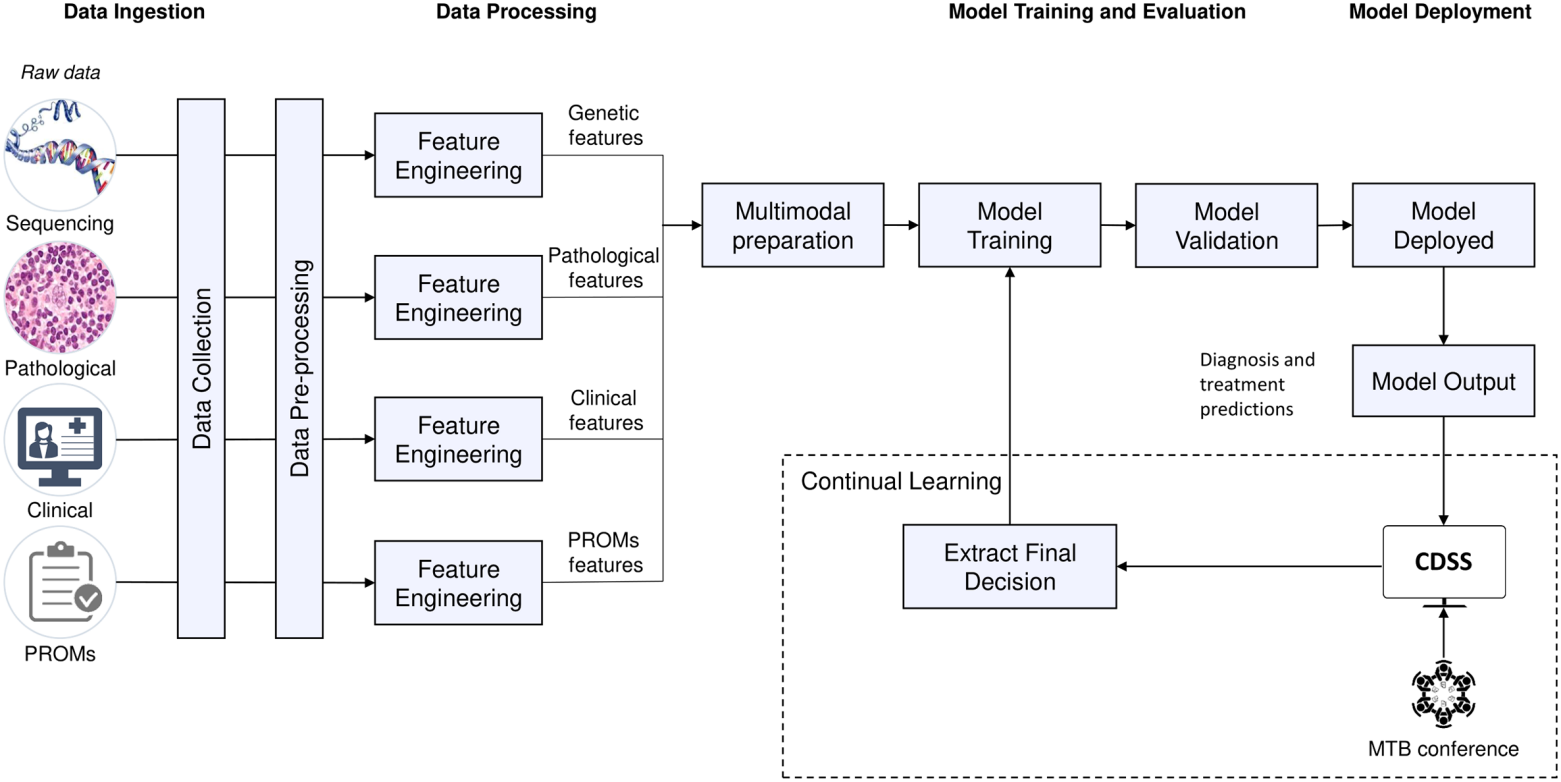
Potential machine learning pipeline.

A UML Activity diagram was created to show the flow of control in the system (**Figure 4**). It contains *actions*, notated as round-cornered rectangles, *objects*, notated as rectangles, and *controls*, which are nodes used to coordinate the flows. The controls in this diagram are the *initial node* (solid circle), *decision node* (diamond-shaped symbol), *fork node* (first line segment), *join node* (second line segment), and *activity final node* (solid circle with a hollow circle inside). Technically, one of the main purposes of the system is to deploy machine learning models to provide recommendations, and we suggest a pipeline to ingest multimodal data and deploy predictive models at point of care (**Figure 5**).

1. Data is collected and preprocessed independently due to data heterogeneity, turning data into a valid format for further processing for data-driven reasoning.
2. Each data input goes through a feature engineering process which enriches the high-dimensional vector representation of the knowledge expressed in identified and relevant published research. To avoid the curse of dimensionality (65), dimension reduction is continuously used, and the correlation between top contributing features is likewise continuously monitored for possible feature elimination to help optimise downstream prediction performance. In this way, the balance between an adequate model with good quantitative performance and an overfitted model that does not generalise well is kept.
3. Multimodal preparation will depend on the selected strategy. In early fusion, unimodal features can be concatenated and used to train one model. In late fusion, each unimodal set of features is used in different models and then the learned features are aggregated. In hybrid fusion, unimodal features are processed before the fusion and may create intermodal features (66).
4. The machine learning model, to be implemented in Python 3.9, is trained and tested using supervised learning for the prediction tasks and unsupervised learning for the clustering of relevant published articles.
5. The machine learning model is validated by assessing its performance with evaluation metrics. Since the cost of false positives and false negatives is low, the oft-used F1 score need not be employed. We will be following the TRIPOD checklist, in which AUC-ROC is the preferred quantitative measure and any splitting into folds for validation are based on time, not stratified by any other means, again adhering to the checklist constraints.
6. The model is deployed offline (batch learning) and online (online learning) in the system.
7. The model output provides predictions of the clinical course (e.g., high probability of treatment refractoriness or early relapse) intended to support the clinical decision-making with respect to treatment selection or adaptation.
8. This final decision will be saved in the system, together with the clinical outcome derived from the decision (after a period of time, contingent on the type of decision). The system will use the decision and clinical outcome to feed the model and improve its performance over time (continual learning).

**Figure 6:**
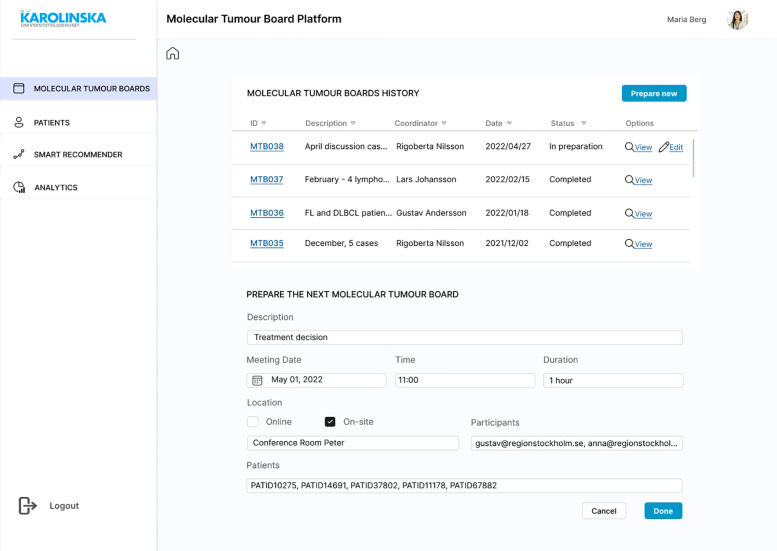
Mock-up screen. MTB history list and “Prepare new” form completed.

**Figure 7:**
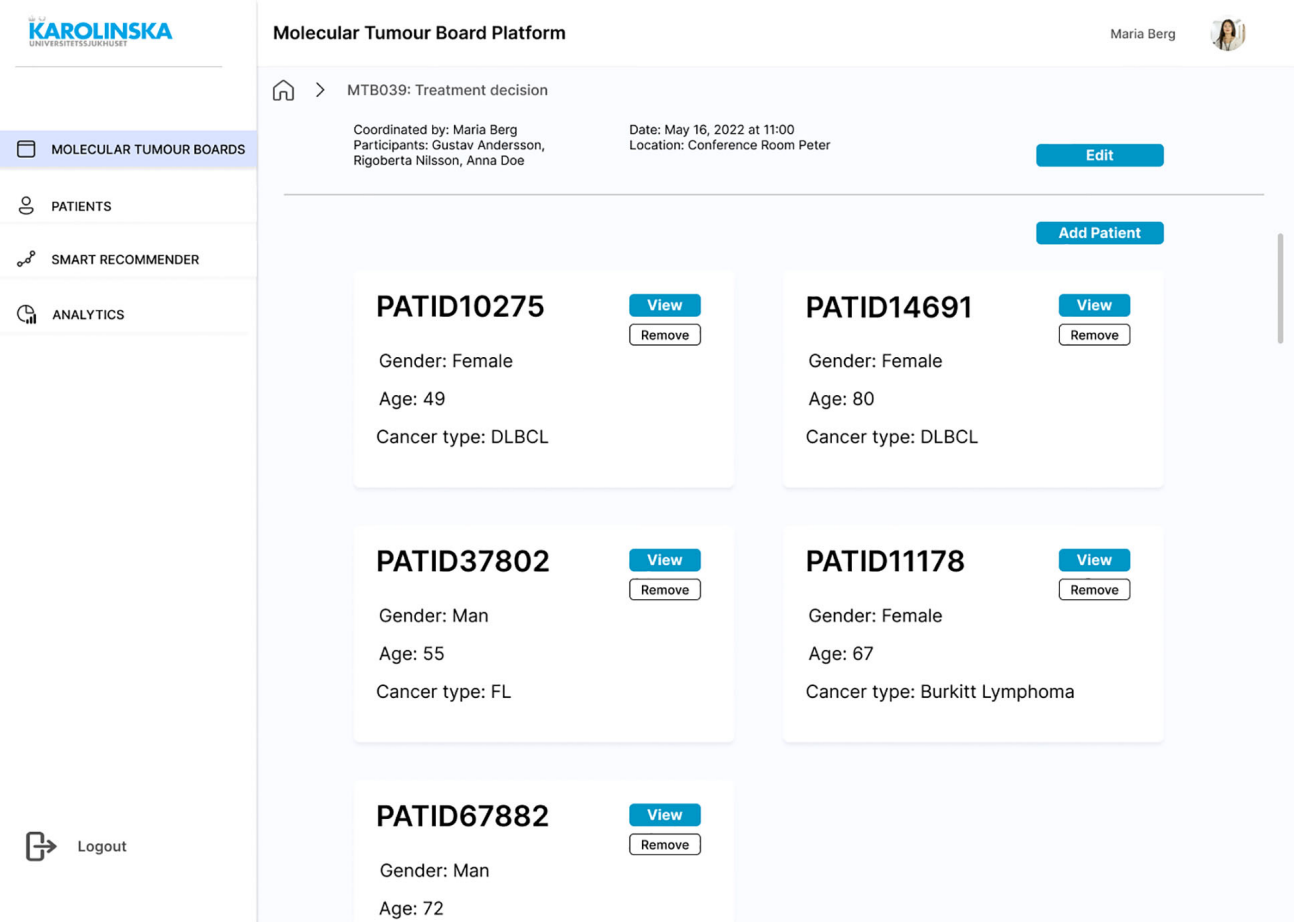
Mock-up screen. MTB overview with a list of five new patient cases to discuss.

A mock-up design was created to complete the requirements engineering process and the Evaluation stage of the DSRM. The first screen is the login page to ensure authenticity. Each registered user will have different access permissions depending on their role to comply with requirements SR23 and SR30. Once the MTB organiser is successfully logged in, the list of past MTBs is displayed, where the outcomes of the discussions will be saved and made available for review, if needed (**Figure 6**). A new board can be scheduled with a brief description of the session, meeting date and time, location, participants, and the identification numbers (IDs) of patients that need to be discussed (**Figure 6**). Once the board is scheduled, it can be opened and see a brief overview of the MTB and a few details about each patient (**Figure 7**). The patient ID has automatically fetched patient information from the HISs (SR07, SR15, SR22). This is the starting page of any MTB meeting, and participants will return to it at the beginning of each patient discussion. If for any reason, there is no time to complete all the patients or there is room for more discussions, the user can remove or add patient cases. This page is intended to be seen by any invited attendee at any time. Therefore, clinicians would have access to all the information before the meeting (SR09), understand the clinical context in advance, and conduct any required investigation.

**Figure 8:**
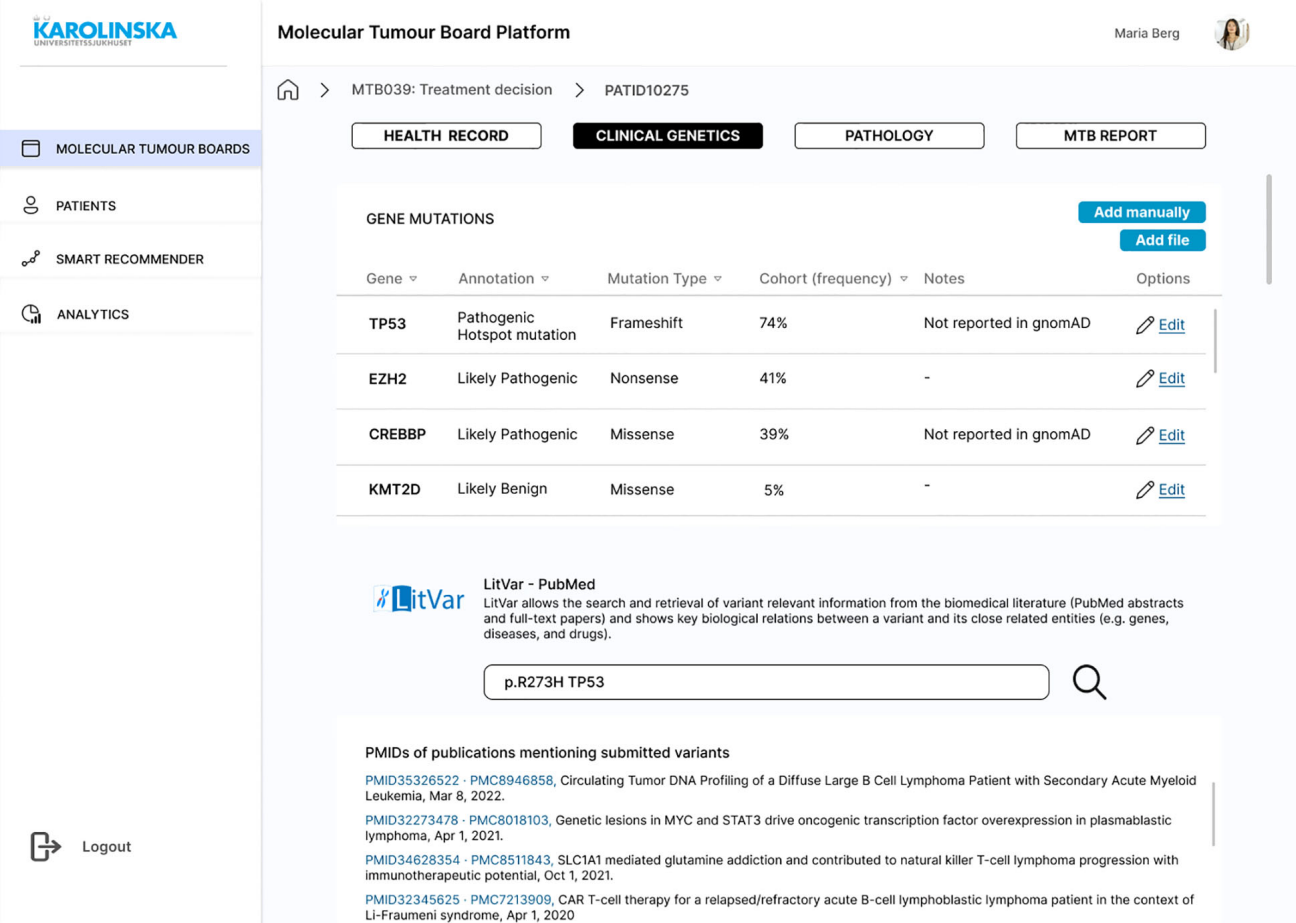
Mock-up screen. Clinical genetics information and LitVar feature.

**Figure 9:**
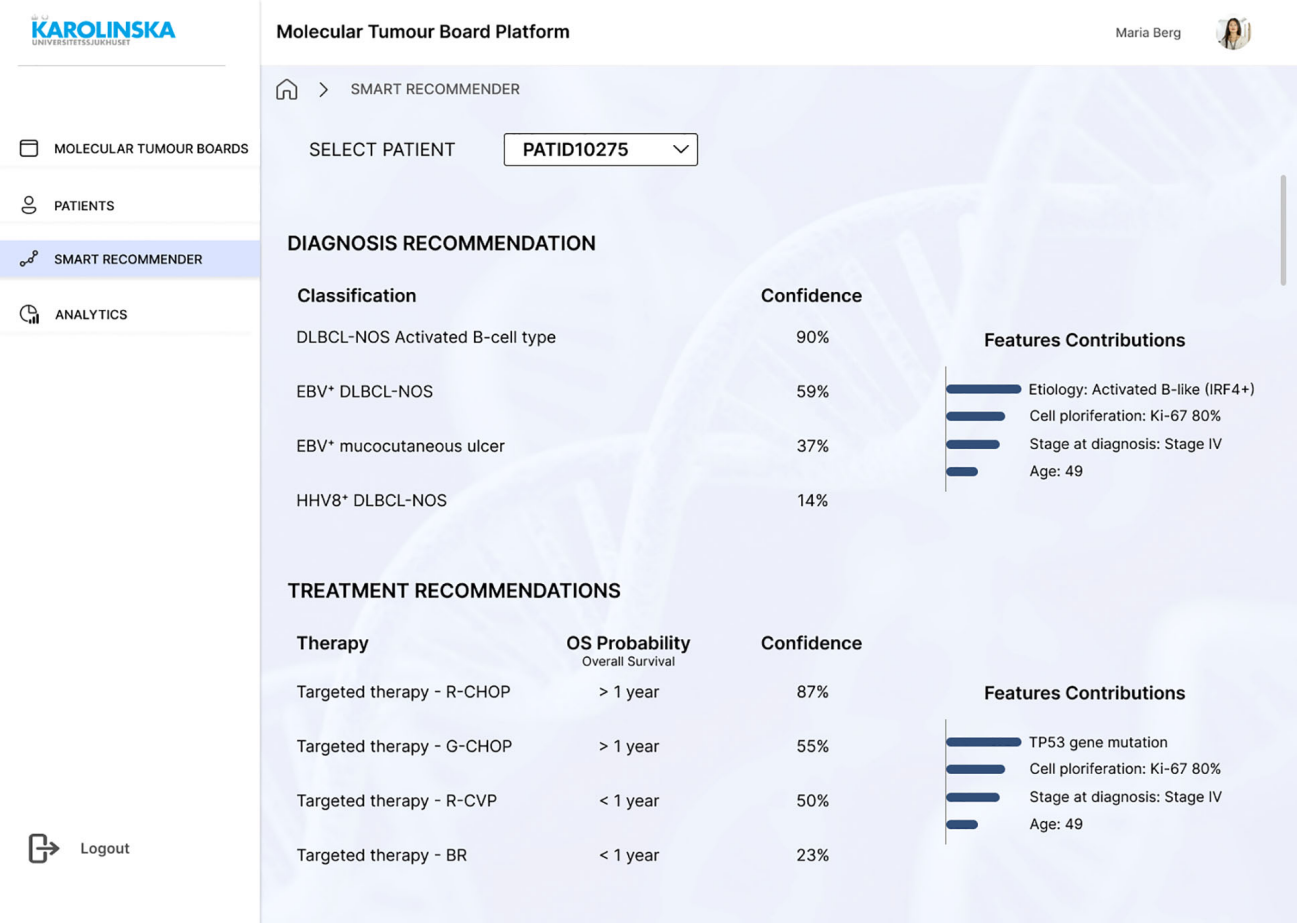
Mock-up screen. “Smart Recommender” giving recommendations for the patient case under discussion.

The patient view consists of four primary tabs: Health Record, Clinical Genetics, Pathology, and MTB Report (**Figure 8**). In Health Record, clinical data (such as chief compliant, history of present illness, past medical history, laboratory test results, and PROMs) are displayed (SR01, SR02). In Clinical Genetics, a list of gene mutations is displayed with their annotation, mutation type, and frequency. Since this information would come from another system that generates a CSV file, the file could be uploaded manually (SR03). In the same tab, the LitVAR feature is included (**Figure 8**), so clinicians write gene variants and retrieve variant relevant information from biomedical literature in PubMed. In Pathology, immunohistochemical staining or other tissue-related slides and written reports with the interpretation are available (SR05). In the MTB Report, the user can register in free text boxes what has been discussed and the consensus, select in a dropdown menu which is the decided diagnosis and treatment, and save the results as a PDF file. Ideally, the report would be exported into the EHR, so the assigned oncologist and physician can access it (SR11). The last screen consists of the “Smart Recommender” feature, which displays the output of machine learning models with diagnosis and treatment recommendations (**Figure 9**). The layout includes the level of confidence and the feature contributions (SR12, SR13).

This mock-up was designed to be user-friendly and intuitive and based on the clinical needs identified (SR24-SR26). Since the mock-up was constructed to validate requirements and if it could achieve the intended tasks, usability was not the main focus of the design. Hence, this first design did not consider all the usability heuristics from Nielsen, as stated in requirement SR27. Ultimately, since the mock-up represented one scenario, it did not include the view of the “Analytics” page, which was included in the left side menu. This would be the page the researchers might have access to, where they could see a dashboard with de-identified data for conducting research studies. The remaining requirements, such as those related to the data warehouse, performance, security, and legal requirements, were not included in the mock-up, as it only represented the user interface.

### 3.5 Requirements validation

Three formal interviews were conducted with clinicians who had participated in the first round of interviews, and one person who had not (see **Supplementary Materials**). In addition, a health informatician oversaw the whole interview process and reviewed its purpose and goals. The participants on the whole demonstrated that their user needs were satisfied. In all cases, interviewees agreed that the solution and the corresponding CDSS are purposeful and something they could certainly see and use in the near future. The results of the AI-based recommendations in the “Smart Recommender” screen in the mock-up (**Figure 9**) were appreciated by the clinicians. The participants were unanimous in the view that this information could help in the meetings. However, as it happened in the initial semi-structured interviews, one interviewee wondered that once the AI is implemented, they might not have anything to discuss. Conversely, another participant featured that clinicians are the decision-makers and AI is just a useful tool that can help, but not decide. Despite these concerns, all participants considered this the future, and a desirable one. One consideration from an interviewee was that in the future, we are going to have several targeted therapies that are directed to patients who have specific gene mutations. Those genetic aberrations would then need to be put in the context of patient characteristics and histology, where MTB would be very much helped by AI. The following requirements were added in the second round of interviews.

- Information about the type of tissue that is available in the archive shall be displayed in the UI. Samples such as biopsies, frozen sections, and biobanking material, are stored for years and can be further analysed, if needed. For research purposes, this information would be highly helpful to have the complete view of a lymphoma patient.
- Pathology panels, such as B-cell or T-cell panels in lymphoma, shall be displayed in the system. In the validation session, pathologists explained that clinicians usually do not understand fully what the pathology slides mean. Instead, panels are easier to look and a more efficient way to understand the results without extra explanations.
- More information about each gene mutation, whether it is tumour tissue or normal control, shall be displayed. One participant suggested that this extra information could be added in a new column, or either having two separated lists.
- More figures and tables shall be displayed in the system. One participant suggested to add figures and tables to show the progression of the patient. Another individual stated that the same information about genetic mutations could also be displayed graphically, because it will be more intuitive to communicate.
- Literature review shall be filtered by diagnostic, treatment or prognostic relevance. Therefore, instead of just searching for genetic variants in the LitVar feature, they should be able to search for more specific publications, depending on which kind of publications they want to find.
- The system shall provide risk assessments based on the multiple data and AI. One participant suggested that this would help to put the treatment recommendation in context.

## 4 Discussion

The scoping review results allowed to fix a preliminary list of general requirements. The thematic analysis of the transcripts was crucial to understand the clinical problem better, identify the specific needs, and design the proposed solution. The complete requirements specification consisted of 43 functional and non-functional requirements. The scoping review results also suggested that the design, development, and implementation of multimodal data-based systems are all very complex. Several studies highlighted important technical requirements that systems in this context of precision oncology, AI, and decision-making support in clinical practice should consider. For example, many articles indicated that MTBs need supporting and scalable informatics tools (1, 27). From the outset, the overall problem was apparent—there is a lack of tools to efficiently support the work of MTB participants during their meetings—but the specific root causes were unknown. After the interviews and thematic analysis from the transcripts, more detailed issues about the processes and tools, challenges, and user needs were identified. This study step was the most important because the participants were clinicians (haematologists, geneticist, bioinformatician) and an experienced data expert, whose conversations allowed for the understanding of their workflow and experiences, and whose involvement was crucial to designing a successful solution. The purported introduction of our solution was also reviewed and consolidated by a health informatician.

The first interesting finding was the need to enhance not only the clinical interpretation of patient data but the end-to-end decision-making process, from the identification of complex cases to the preparation of the meeting and reporting of the final decision. While the main problem was dealing with a significant amount of data, the results suggested that the meeting preparation is a key process to consider and there is room for improvement, in agreement with the literature (67) in which reports found that a large amount of time is required during the MTB preparation for patient data collection and literature search. In the current workflow (**Figure 1**), the different MTB experts arrive at the meeting without prior knowledge about the clinical context or pathology data, for example, if the expert is a geneticist. This fact increases the discussion time during the meeting since all the participants must present their data and findings and then interpret the results. If they had a solution where they could see and share data before the meeting, it would increase the efficiency of the patient case discussion and probably, discuss more patients in the same session. Another debating point was the data collection for the MTB meeting. In the current workflow, the MTB coordinator asks the other MTB specialists to send the patient data over in a manual process, and the coordinator includes the information as a slides presentation. This is a time-consuming task and prone to errors, suggesting some degree of automation would be desirable. A commercial solution from Roche, NAVIFY^®^ Tumour Board, was also designed to improve the MTB preparation process (68). Even though other studies define digital tools for MTBs using AI, they all focus on specific cancer types, such as lung cancer (43), metastatic melanoma, metastatic epithelial ovarian cancer, and acute myeloid leukemia (46), or prostate cancer (69). This study did not find any attempt to develop an AI-based CDSS specifically for lymphoma. Digital solutions without AI are, however, of potential interest too (32, 70, 71).

Our study showed that AI could contribute to efficient group discussions in MTB meetings because clinicians themselves struggle to interpret all data together and predict single patient outcomes. These results match those observed in earlier studies. Macchia et al. (72) developed a “MTB Virtual Assistant”, which used NLP in clinical diagnostic reports to transform the information into structured data and applied models to assess the disease staging. IBM launched IBM Watson for Oncology, a CDSS which uses machine learning to learn from clinical guidelines and recommend a treatment plan (43–45). Instead, the current study suggested a recommender system based on continual learning, which could progressively learn from their mistakes and improve their performance. Therefore, this study supports previous observations that pointed to continual learning AI as a potential application in CDSSs (25, 73). Still, there are a lot of challenges before we could see continual learning CDSSs in the real clinical setting. For instance, there is a risk of *catastrophic forgetting*, in which the new data interferes with what the model has already learned (25, 74). Additionally, Jacobs et al. (75) conducted a factorial experiment with clinicians in which the results suggested that incorrect recommendations might adversely impact the treatment decision of clinicians, even if they are supported with explanations. They also found that prior experience with machine learning may influence the use of recommendations in their decision-making. Therefore, it is of great importance to design learning models with significant levels of trust and take into account the familiarity of clinicians with machine learning.

The proposed CDSS concurs with several studies (20, 76, 77) which indicated that multimodal AI presents great opportunities for patient stratification and precision medicine. Consistent with the literature, this research found that a robust data warehouse is requisite to enable data extraction, pre-processing, processing, model deployment, and data storage and security. Recent efforts in Karolinska University Hospital may allow the implementation of the proposed solution. In 2021, the hospital upgraded the existing data warehouse with a clinical data repository based on the standard openEHR and is developing a scalable “Storage and Computation” solution, and integration platform (78). It is therefore likely that such efforts may benefit the implementation of the proposed solution for MTBs in this study, since Karolinska aims to include EMR data, waveform data, imaging, test-based diagnostics, and omics data, for clinical decision support, quality management benchmarking, precision medicine, and research.

Security and legal requirements play an essential role in this landscape and may determine the implementation of precision oncology in clinical practice. Even though the results highlighted a list of EU regulations to follow, there are no clearly stated regulations that address the legal and ethical problems that AI may cause (79). MDR includes a list of classification rules which explain what medical device fall under each classification, based on the risks associated, the technical design, and how it is manufactured. There are four product classes from low to high risk: Class I, Class IIa, Class IIb, and Class III. MDR Rule 11 applies to medical software (Software as a Medical Device, SaMD): *“Software intended to provide information which is used to take decisions with diagnosis or therapeutic purposes is classified as class IIa, except if such decisions have an impact that may cause: death or an irreversible deterioration of a person’s state of health, in which case it is in class III; or a serious deterioration of a person’s state of health or a surgical intervention, in which case it is classified as class IIb. Software intended to monitor physiological processes is classified as class IIa, except if it is intended for monitoring of vital physiological parameters, where the nature of variations of those parameters is such that it could result in immediate danger to the patient, in which case it is classified as class IIb.”* (80). Since the proposed system is intended to provide information used to make decisions with diagnosis and therapeutic purposes, it would likely have to follow requirements for Class IIa devices. Medical devices must be labelled with the CE marking under the MDR to be freely traded in the European Economic Area (EEA). This fact may rise issues for AI-based systems applying continual learning, since the system that once was CE marked, keeps learning and may experience behavioural changes (81). Therefore, the system performance is altered post approval.

Our study is based on the needs of actual end-users, which most likely increases the chances of a future implementation of the solution. Due to the study setting, only a few prospective end-users were interviewed. The participant selection included MTB specialists and IT specialists. While we consider the four MTB specialists as providing us with a great amount of information and insights, it would have been interesting and possibly fruitful to interview more than one IT specialist. Surprisingly, few related studies involve clinicians. For example, Halfmann et al. (67) in their study used questionnaires and interviews as the methods to gather information about requirements, workflows, weaknesses, and potential functionalities from MTB organisers. They performed a task analysis and used the results to develop a layout. Instead, Buechner et al. (82) conducted group interviews. While focus groups would probably have been a more effective solution in this study, and they may provide different insights from the discussion between participants, it may reduce the freedom of each participant to express their thoughts and be less focused than an individual interview.

The selection of participants directly impacted the results of the study. In the first round of interviews, haematologists, a geneticist, and a bioinformatician participated, even though pathologists are also involved in MTBs. Then, for the end-user validation process, both clinicians who had participated and clinicians who had not, including a pathologist, were involved. In this way, we ensured that the requirements were not only validated with the users who stated them, but also with other clinicians. As a result, the pathologist asked for some data requirements that were not visible in the digital mock-up, such as having pathology panels or information about the availability of tissues in the archive.

Our scoping review is limited in the sense that some technical terms were only used for IEEExplore, but not for Web of Science. Due to the limited time and the narrow study setting, a small sample of stakeholders was interviewed. While we consider that four MTB specialists provided me with a great amount of information and insights for the aim of this study, it would have been interesting to interview more than one IT specialist. Requirements engineering is a slow process, sometimes referred to as addressing a ‘wicked’ problem that cannot even be properly described, let alone adequately addressed, in full. The Volere Requirements Specification Template (53) in particular consists of a 27-item checklist for requirements. Our study was limited at this stage only to include requirement type and descriptions of each requirement, reflecting the early stage of development in the system life cycle. Future efforts will consider the full template. An additional limitation was that the mock-up design only simulated one scenario. The system requirements included more functionalities than those represented in the mock-up. The MTBs considered were those of the BioLymph project, and no existing conferences for other types of cancer at Karolinska University Hospital were considered. Hence, there was no surgeon role in the MTBs considered, for example. Finally, more technical requirements regarding data warehousing, computational performance, and security were not considered in the evaluation process. This would have required other types of professionals, all of which are available in the environment studied, and so should be consulted in any future work. System architects must also explore the integration of the proposed CDSS and existing health informatics systems, taking into account international standards, and avoiding 1:1 proprietary integrations. Researchers with experience in data science and AI should work on building interoperable databases, performing feature engineering processes with the different data modalities, exploring federated architectures and other means to secure data management, investigating scalability, and developing machine learning algorithms. Such algorithms will then require rigorous studies and randomised clinical trials (76). Considerably more work will need to be done to determine the feasibility of such development and implementation in the hospital setting. Further research needs to examine algorithms more closely, including continual learning, and explore how to maintain the system over time. Other use cases with machine learning that could also add value to the meeting are algorithms for early identification of high-risk patients and risk stratification.

We envisage researchers and developers with experience of data science and AI developing machine learning algorithms for and with clinicians. Their tasks then include building interoperable databases, turning important clinical variables into feature sets for algorithms, and implementing federated architectures. All of these tasks will also shape validation, ultimately in the form of randomised clinical trials (76). Besides assisting MTBs in the ways we have described, meta-properties of MTBs will be examined closely and used for internal validation purposes, such as adjusting the balance between specialist competencies in meetings in accordance with goals and with metadata on decisions and discussions automatically monitored and continuously analysed. Various large and long-term initiatives in the ecosystem constituted by the local hospital and research institute are directly supporting such ambitions. Feasibility studies and the continual learning on the part of the AI systems employed will then be investigated in the hospital setting. New use cases that would make MTBs even more useful include machine learning for the early identification of high-risk patients and risk stratification of patient cohorts.

## 5 Conclusion

Based on an extensive qualitative analysis, this study introduced a consolidated proof of concept which demonstrated that a Web-based information system for visualising relevant lymphoma patient data and giving AI-based diagnosis and treatment recommendations can support the end-to-end clinical decision-making process of MTBs at Karolinska University Hospital. The results include a list of 43 requirements elicited from main stakeholders, MTB specialists, and an IT expert, which together provided advice and guidelines for creating a mock-up design. Interactive sessions with real end-users allowed for a requirements validation, and the identification of six additional requirements. That validation consolidated that the proposed system could meet the challenges from clinicians of conducting efficient meetings, with decisions taken based on complex molecular data.

These results add to the rapidly expanding field of precision oncology in the form of a blueprint of a holistic system that contributes to make faster and more accurate decisions, reduce error-prone process steps, and further improve data quality. Clinicians using our decision support system would distill the most important features from large datasets and achieve accurate lymphoma patient stratification, which leads to better clinical outcomes, subject to further randomised clinical trials. This work could then be extended further, to select other cancer types and institutions. Overall, our study contributes to carefully adding learning approaches to the field of Translational Bioinformatics, by optimising medical and genomic data for decisions that directly impact the quality of life of patients.

## Data availability statement

The original contributions presented in the study are included in the article/**Supplementary Material**. Further inquiries can be directed to the corresponding author.

## Ethics statement

The prospective cohort study used as a basis for the here reported work has been ethically approved (Dnr 2017/2538-31) and this approval includes development of routines related to precision medicine and individual patient data interpretation in clinical care. No further ethical approval was required for our study, as it is not depending on real patient data.

## Author contributions

NR and MB wrote the manuscript. All other authors were stakeholders involved in the validation or refinement of the requirements, and also helped improve the manuscript. All authors contributed to the article and approved the submitted version.

## Funding

Parts of this work were funded by Stockholm County Council (grant No 20190494) and the research funds of Radiumhemmet (grant No 214153). In addition, TW was supported by Region Stockholm (clinical postdoctoral appointment). The authors will receive funds for open access publication fees from KTH Royal Institute of Technology.

## Acknowledgments

We thank Richard Rosenquist Brandell and Hassan Foroughi Asl for important assistance.

## Conflict of interest

The authors declare that the research was conducted in the absence of any commercial or financial relationships that could be construed as a potential conflict of interest.

## Publisher’s note

All claims expressed in this article are solely those of the authors and do not necessarily represent those of their affiliated organizations, or those of the publisher, the editors and the reviewers. Any product that may be evaluated in this article, or claim that may be made by its manufacturer, is not guaranteed or endorsed by the publisher.
