# Supplementary material for: Data-driven support to decision-making in molecular tumour boards for lymphoma: A design science approach
Source: Front Oncol. 2022 Nov 15;12:984021. doi: 10.3389/fonc.2022.984021 (PMC9705761; doi:10.3389/fonc.2022.984021)
Supplement: Supplementary file 1 [file DataSheet_1.pdf]

Supplementary Material

1 SUPPLEMENTARY DATA

1.1 Template for the Informed Consent Form

INTERVIEW INFORMED CONSENT FORM

Participant Name: xx xxx

Interview Date: xx/xx/xx

Project Title:
Multimodal Data Interpretation to Support Clinical Decision-Making in Precision Oncology: A Conceptual Design for Molecular Tumour Board Meetings

Researcher Name/ Author:
Núria Rodríguez Ruiz, nuria.rodriguez.ruiz@stud.ki.se

Description of the project:
The research project aims to conceptually design a clinical decision support system to facilitate the multimodal data interpretation in molecular tumour board meetings for lymphoma patients at Karolinska University Hospital. The purpose of this document is to specify the terms of my participation in the project through being interviewed.

I understand that...

- I voluntarily participate in the interview and research study.
- The interview will last 45 minutes approximately.
- The interview will be audio-recorded.
- The audio will be transcribed for further data analysis.
- My confidentiality will be guarantee in the transcripts and in the thesis report.
- Transcripts will be analysed through thematic content analysis.
- I can withdraw permission to use my interview data after the interview. In that case, the recording and transcripts of my interview will be deleted.
- The research author may publish quotations by me in the thesis report.

By signing this form, I agree to the statements indicated above

Signature of participant	Date

Signature of researcher	Date

2 SUPPLEMENTARY TABLES AND FIGURES

2.1 Tables

Table S1. Overview of the selected studies.

Category	Number of Studies
**Year**	
2022	4
2021	12
2020	11
2019	5
2018	4
2017	2
**Country**	
Germany	7
US	7
Italy	3
Republic of Korea	3
China	2
Greece	2
Sweden	2
Switzerland	2
Belgium, Bulgaria, Canada, Chile, France India, Mexico, the Netherlands, UK, United Arab Emirates	1 (for each country)

Table S2. Preliminary requirements as a result of the scoping review.

ID	Requirement	Ref.
**Data Warehouse**		
LR01	Computational data analysis pipelines shall cover the analysis from raw data to reporting.	[1]
LR02	The system shall enable a harmonised interpretation of genetic data by a structured and standardised process.	[2] [3] [4]
LR03	Data from EHR shall be standardised to enable automatic integration processes.	[5] [4]
LR04	ETL (Extract, Transform, Load) workflow shall be automated.	[5] [3] [6]
LR05	System design shall allow further maintenance and support. Standardisation is a success factor.	[4] [7]
LR06	Data shall be stored in interoperable and interrogatable databases.	[8] [6] [9]

LR07	The system shall enable real-time data collection.	[10]
LR08	PROMs shall be integrated within the EHR system.	[11]
LR09	Data analysis workflow shall be an efficient process (distributed computing such as high-performance clusters or cloud engines).	[12] [1]
LR10	Data warehouse shall store raw data and metadata.	[13]
LR11	Data warehouse shall integrate data from different types or modalities.	[14]
LR12	A database shall be created to store information on biomarkers such as gene, aberration, drug, target, availability, tumour type, and PubMed ID.	[15]
LR13	Tables shall be maintained with relational SQL databases.	[16]
LR14	Data warehouse shall support HL7/FHIR as healthcare informatics standards and the connection to statistical tools such as R and Python.	[13]
LR15	Data warehouse shall support the connection between data and AI frameworks such as Tensorflow, Keras, or PyTorch.	[13]
**Features**		
LR16	Data shall be presented in a user-friendly format before the time of the MTB meeting.	[12]
LR17	A CDSS for MTBs shall include metadata about the quality of the data sources, the curation process and all the steps of the integration pipeline including tools, versions and settings.	[1] [17] [9]
LR18	The system shall facilitate collaboration.	[12]
LR19	The system shall enable a sharing responsibility of final conclusions with specialists in a collaborative manner.	[15]
LR20	The system shall allow multidisciplinary access and use.	[12]
**MTB Report**		
LR21	The system shall generate a standardised report with a summary of the findings and overall recommendation.	[18] [1]
LR22	The report shall include a list of clinical trial opportunities based on molecular profile of the tumour.	[18] [12] [1] [16]
LR23	The report shall include a list of therapy recommendations.	[1]
LR24	The report shall include a tailored treatment plan.	[14]
LR25	MTB recommendations shall be clear.	[18]
**Principles and Ethics**		
LR26	Computational pipeline shall be stable, robust, reproducible, and traceable.	[1] [11]
LR27	Machine learning models shall not be black-boxes and must consider explainability approaches.	[6] [11] [7]
LR28	The system shall follow the FAIR guiding principles for scientific data.	[17] [11] [19] [20] [13]
LR29	The system shall implement an audit trial of the data the decisions were based on.	[17] [11]
LR30	The system shall ensure data quality.	[19]
LR31	A CDSS shall be transparent.	[11]
LR32	The system shall be scalable.	[21]

LR33	The use of AI models shall be in line with privacy laws.	[20]
**Security and Privacy**		
LR34	The system shall preserve patient privacy and security of sensitive data.	[1] [12] [3] [7]
LR35	Exchange data between institutions shall be in compliance with data protection regulations.	[22]
LR36	The system shall have a security framework to restrict authorisation and set authentication schemes to allow access only with valid credentials.	[1] [3] [19]
**User Interface**		
LR37	The system shall have a modern user interface, tailored to the needs of the clinical initiative.	[15]
LR38	The system shall allow the interactions with the database through a portal.	[16]
LR39	The user interface design shall enhance the usability in an efficient and effective way.	[11]
LR40	The UI design shall follow usability principles (e.g., General Usability Heuristics from Jakob Nielsen)	[11]

Table S3. Interview Guide for MTB Specialists.

**Purpose**	**Questions**
Researcher introduction	• Name, background, and interests.<br>• Brief introduction of the study.<br>• Remind the statements in the informed consent form.
Interviewee introduction	• Please, tell me about yourself and your current occupation.<br>• What is your interest in Molecular Tumour Boards?<br>• How important do you think they are for *current* decision-making and in the *future*?
Understanding MTBs	• Which specialists are taking part in MTB discussion in BioLymph, and which is your role in there?<br>• How do you prepare for the meeting? Is there a coordinator role who collects all this data before the meeting?

Information flow	• From your speciality perspective, what should molecular and non-molecular data be used to achieve a fruitful discussion?<br>• Could you please list the data type and data sources?<br>• Which information should the outcome of the MTB include?<br>• Where and with which format it is reported?
Clinical interpretation	• MTB can make biological classifications or clinical classifications for identification of variant-drug or gene-drug associations. At which levels do you need to classify the results in the BioLymph study?<br>• Which sources/databases do you look at?
Clinical challenges	• What challenges are you encountering during the process, from the preparation of the MTB meeting to the generation of the conclusion?
Closing	• Are you aware of the existence of IT systems to support MTB meetings? Have you considered using them and why?<br>• How do you imagine an ideal solution to improve MTBs?<br>• Is there anything more you would like to add that you consider valuable for the scope of this study?

Table S4. Interview Guide for IT Specialist.

**Purpose**	**Questions**
Researcher introduction	• Name, background, and interests.<br>• Brief introduction of the study.<br>• Remind the statements in the informed consent form.
Interviewee introduction	• Please, tell me about yourself and your current occupation.<br>• What is your interest in Molecular Tumour Boards?<br>• How important do you think they are for *current* decision-making and in the *future*?

Multimodal and AI-systems awareness	• Do you know if there is any system in the hospital to support the clinical decision-making of any MTB?<br>• What would you think about implementing a system that integrates multiple resource data?<br>• Do you think it could be based on AI methods to enable predictive analysis?
IT challenges	• What challenges can you see in the implementation of such a system in the hospital?<br>• Could you please list the data type and data sources?<br>• Effective implementation of AI technologies requires computational power resources. Instead of local processing, do you think a cloud computing alternative can be considered?
Closing	• What elements would you see in an ideal data infrastructure that would support this implementation?<br>• How do you imagine an ideal solution to improve MTBs?<br>• Is there anything more you would like to add that you consider valuable for the scope of this study?

Table S5. Participants in the semi-structured interviews (Define solution requirements Phase).

Alias	Role	Group	DateTime	Duration
Bioinformatician	Bioinformatics Scientist at KI, experienced in cancer genetics.	MTB	March 2	38 min
Haematologist	Resident in Haematology at Karolinska and affiliated researcher at KI (lymphoma).	MTB	March 2	31 min
Geneticist	Clinical geneticist at Karolinska and affiliated researcher at KI (haematological malignancies).	MTB	March 2	44 min
Oncologist	Clinical doctor at Karolinska and professor in haematology-oncology at KI (lymphoma).	MTB	March 3	43 min
Data Expert	PhD, Head of Data, and extensive experience in data infrastructures in life sciences.	IT	March 14	30 min

Table S6. Participants in the semi-structured interviews (Evaluation Phase).

Alias	Role	Prior Participation
Haematologist	Resident in Haematology at Karolinska and research affiliated (lymphoma).	Yes
Oncologist	Clinical doctor at Karolinska and professor in haematology oncology at KI.	Yes
Pathologist	Pathologist at Karolinska and research affiliated (lymphoma).	No

Table S7. Thematic analysis of interview data.

**THEME 1**	**MTB for lymphomas**
**Subtheme:**	**Current situation**
**Codes:**	MTB meetings for lymphoma are on an experimental level still.<br>Meetings are not taking place now because the new techniques are not established in the clinic.<br>The goal is to sit together to try to reach a conclusion on how they can use all the different data.<br>Clinical geneticists are really needed by clinicians since it is very detailed data that they cannot understand the results.<br>Make decisions based on pathogenecity of findings.<br>The current meetings (for research purposes) discuss for four or five patients at a time.<br>The diagnostic workup lasts two weeks approx.
**Subtheme:**	**MTB preparation**
**Codes:**	Preparation of the MTB is done from the clinic side.<br>Preparation involves clinical data, genetic data, and pathological data.<br>One clinician coordinates the meeting and sends patient numbers out to the other specialists.<br>Member of each speciality prepare the results and investigate the findings in knowledge bases.<br>Manual data collection.<br>Patient selection criteria: now the more complex cases.<br>The coordinator adds all the information in a slides presentation.
**Subtheme:**	**MTB relevance**
**Codes:**	Integration of molecular and nonmolecular data.<br>Genetic data help refine the lymphoma subtype diagnosis.<br>MTB will be increasinlgy important as we go along.<br>Educational opportunity.<br>Discussion for one patient may help other patients.<br>It is a very important meeting point.

	More impactful if new technology is used.
	Good for collecting information from complex data and making it presentable for decision-making.
**Subtheme:**	**Stakeholders**
**Codes:**	Multidisciplinary group.
	Clinical geneticists as decision-makers.
	Haematologists or oncologists (both work clinically with lymphoma patients) as decision-makers.
	Haemophatologists as decision-makers.
	Physicians.
	Bioinformaticians as a supporting role to evaluate the results from a data point of view.
	Other stakeholders may be welcome if they are employed by the hospital.
	Everyone working in the hospital involved in the lymphoma patient can attend the conference and listen.
**THEME 2**	**Relevant information for discussion**
**Subtheme:**	**Clinical data**
**Codes:**	Current status of the patient.
	Treatment received and response after treatment.
	Must ensure to put genetic data in its clinical context.
	Tumour information.
	Medical history: age, sex, number of comorbidities, type of comorbidities, performance status, blood test results (show the status of lymphoma and other organ functions), lactate dehydrogenase (LDH) as a prognostic factor, CT or PET CT scan (stage of the disease, spread of disease, one or multiple locations), bone marrow biopsy.
	Extracted from the hospital healthcare information system.
**Subtheme:**	**Genetic data**
**Codes:**	Somatic mutations.
	Novel and actionable genetic variants.
	Translocations for diagnostic and prognostic.
	Genetic data is the core and the new information in MTB discussions. Participants look if it helps in some way.
	NGS of DNA, RNA and protein.
	It helps to confirm the diagnosis, but also to decide which grade a tumour is and to choose the right treatment.
	With very known genetic alterations is easy to interpret both the diagnosis and prognosis.
	With non-common genetic alterations, rare and new, one needs to go through a lot of databases reports and check the meaning.
	MTB discusses the combination of these findings with the other clinical and pathological information.
**Subtheme:**	**Pathology data**
**Codes:**	Staining tests (Immunohistochemical).

	Lymph node biopsy.<br>Pathology reports: morphology of the cells, how they interact
**Subtheme:**	**Metadata**
**Codes:**	Reproducibility and trace decisions.<br>It could add an extra layer of information for the decision-making.<br>It can reduce the number of false positives.<br>Experimental metadata, sample metadata and biomaterial metadata.<br>Metadata from sample collection, library preparation and data processing.<br>Involved software versions.
**THEME 3**	**Involved tools and other solutions**
**Subtheme:**	**Current supporting tools**
**Codes:**	Scout (geneticists software) for sequencing data analysis and automatic and standardised classification which filters missense, noise, and artefacts.<br>PowerPoint for presenting results in the meeting.<br>TakeCare.<br>Occasionally, Cisco for external communication to other hospitals to discuss complex cases.
**Subtheme:**	**Knowledge Bases**
**Codes:**	"gnomAD", database to find normal common variants.<br>"COSMIC", "My Cancer Genome", "oncoKB".<br>Apart from cancer knowledge bases, they look into PubMed.
**Subtheme:**	**Other solutions**
**Codes:**	Tools by Foundation Medicine offers a PDF report.<br>Device that listens to the conversation and provides feedback.<br>Commercial solutions focus on genetic findings.<br>Molecular Tumour Board Portal for solid malignancies
**THEME 4**	**Challenges and Problems**
**Subtheme:**	**Challenge**
**Codes:**	Stratify patients into all the categories in lymphoma in the clinic.<br>It is a difficult process to organise, summarise and make easily available all the data from geneticists.<br>It is difficult to have all this knowledge for one person.<br>It is not possible to use external/commercial solutions to support MTB meetings because they cannot put patient sensitive data (NGS).<br>To make sure the new knowledge reach to patients.<br>The work process needs to be more rapid and smooth for the patient to benefit.<br>The genetic landscape will become even more complex than we already know and it will be hard to understand the many different layers of information.<br>To have the information controlled and updated.<br>Distill the most important features from very complex datasets.
**Subtheme:**	**Problem**
**Codes:**	MTB participants may not take into account a genetic type because they do not know their significance as they do with others.<br>Geneticists and oncologists have a high workload pressure.

	There is all the time new information coming in the literature.
	Data is in different systems. MTB participants do not have access to other specialities' systems, and therefore to their data.
	Knowledge bases are in different way updated. Geneticists need to go through all these databases because new variants appear all the time.
	It is difficult to exchange patient data and information between institutions in Sweden, and outside Sweden.
	Genetic panels are not yet included as a routine clinical test.
	PubMed is not practical.
	There is a mismatch with databases and they are not always updated.
	From the clinician part, there is a lack of understanding of mutations.
	There is a lack of interactive resources.
**THEME 5**	**User needs**
**Subtheme:**	**Characteristics**
**Codes:**	It should be part of the hospital, rather than external system.
	It must be accessible.
	Haematologists, geneticists, pathologists should be able to input their data independently before the meeting.
	A common workspace is needed such as an interactive panel in front of everyone.
	Easy for someone who is not very knowledgeable in genetics.
	It should have an easy way of reviewing the information, self-explanatory, manageable, easily available and easily presented.
	The solution should not take a lot of time with easy cancer cases.
	Help the understanding of clinical relevance of the new biological data.
	To have the answers in less time (more efficient process).
**Subtheme:**	**Function**
**Codes:**	It should have all the different aspects to guide the clinical decision-making.
	It must be updated all the time for the accurate decision-making.
	Automatically retrieval of information from data sources would efficient the process.
	It should read through all the information and compile everything together.
	Integration of genetic data with other kinds of information (clinical and pathology data)
	To be able to enter one or combination of mutations (results of the patient) and get information at diagnostic, prognostic and predictive level.
**Subtheme:**	**Input**
**Codes:**	Mutations in particular subtypes in relation to diagnosis, prognosis and prediction of treatments.
	Molecular characteristics and other information should be available during the diagnostic workup.
	A risk score could guide the experts into choosing a treatment strategy.
	Treatment stratification based on genetic data.
	Data should be good and relevant (quality).
	Well-communicated information.
	Which treatments should choose.

	To include if genetic help to choose a particular therapeutic agent or should not give that therapy.<br>Information must allow to have the complete picture (clinical context).<br>Important information should be lifted up.
**Subtheme:**	**Output**
**Codes:**	MTB conclusion could be exported and reported as an entry in the hospital medical records.<br>Final report should include the confirmation of the lymphoma classification (diagnosis).<br>Final report should include: who participated, when, what has been discussed, which was the result.<br>Participants can write free text in the final report.<br>The outcome of the discussion should be a consensus.<br>The final report could be saved in the solution.
**Subtheme:**	**Privacy**
**Codes:**	Access must be given to MTB participants with confirmed login credentials.<br>Appropriate level of protection for patient data privacy.
**THEME 6**	**Technical considerations**
**Subtheme:**	**Academia vs. Clinic**
**Codes:**	Clinical and academic environment are very different.<br>Systems in clinical routine (production level software) require a more complex setting than systems in the research level.<br>Transfer data back and forth between academic side of the hospital is complex.<br>Production level software need well-planned data flows and workflow.<br>Translational research and clinical routine are two sides not so easy to match.
**Subtheme:**	**Resources**
**Codes:**	System must have an organisation around it that can ensure that the software is maintained and developed properly.<br>For complex computational pipelines, certain types of storage of certain types of processing (GPU, CPU) are needed.<br>Cloud resources are expensive.<br>With local processing, you have the responsibility to keep it run and secure.<br>With local processing, you may need to hire more people.<br>It is important to choose wisely which resources are used and ensure no one can break the system.<br>Hybrid solution may be considered. Local resources first, and add cloud resources to cut the peaks if more storage is needed.<br>The solution must be planned in an economic way.<br>Local resources are beneficial if there is local expertise that can cover the chain of information.
**Subtheme:**	**Keep track of decisions**
**Codes:**	The system must keep track of all the decisions.<br>Software in the hospital needs someone who is responsible for its maintenance.<br>It is important to have the chain down of responsibility (track down when and for who the decision was made).

**Subtheme:**	**Legal considerations**
**Codes:**	Software in the hospital needs legal agreements.
	Cloud resources are doable if we have the right legal agreements for that.
	Data processing agreements are needed if it is done by the academia.
	Patients must give their consent.
**Subtheme:**	**IT benefits**
**Codes:**	Systems in MTB make pressure on geneticists go down and reduce errors.
	Automatic information retrieval reduce errors.
**THEME 7**	**Artificial Intelligence**
**Subtheme:**	**Black box**
**Codes:**	AI is a black box.
	AI as a black box is blocking its clinical use.
**Subtheme:**	**Future of AI**
**Codes:**	AI could give the next step in MTB conferences.
	AI could indicate the risk, the prognosis and best treatment based on the genetic aberrations the geneticists have identified.
	MTB will need machine informed or AI informed decision-making.
**Subtheme:**	**Potential**
**Codes:**	AI could reduce false positives.
	AI could reduce false negatives.
	AI to narrow the gap between over reporting and under reporting.
	AI has a lot of potential to help us.
	MTB could be faster and more accurate with AI.
	With AI we can assess more complex data inputs than before.
	AI can distill very complex data and provide alternative decisions and predictive outcomes of those.

2.2 Figures

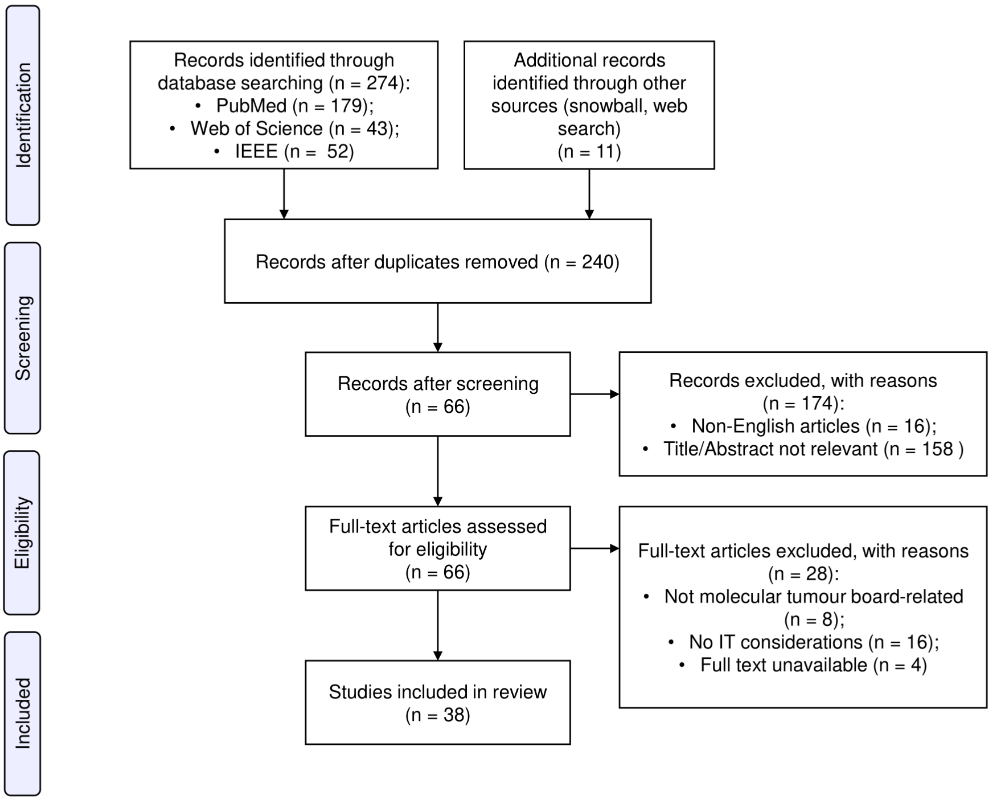

Figure S1. PRISMA flow chart of the scoping review.

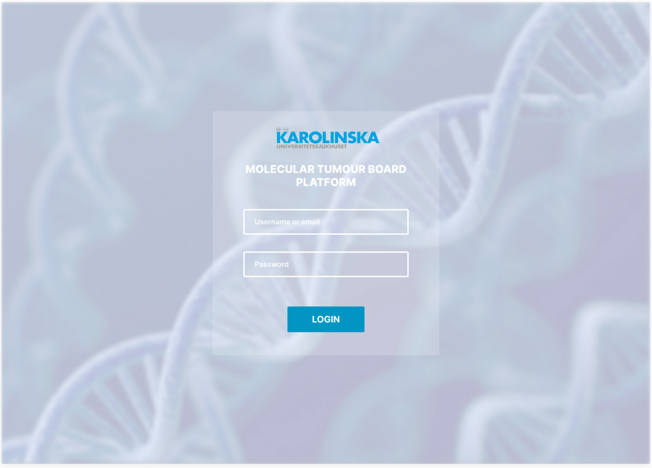

Figure S2. Login.

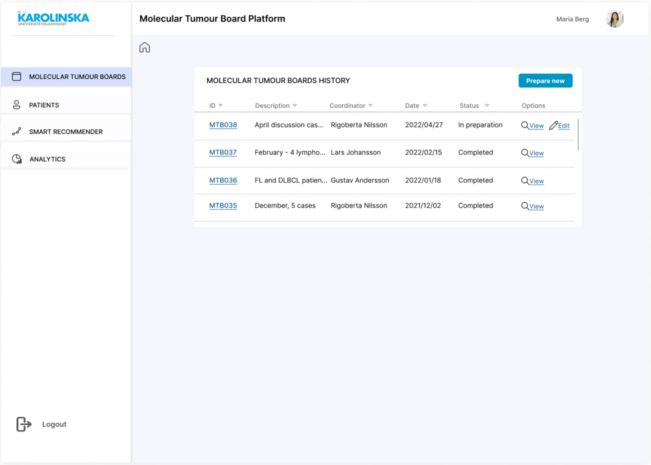

Figure S3. Home.

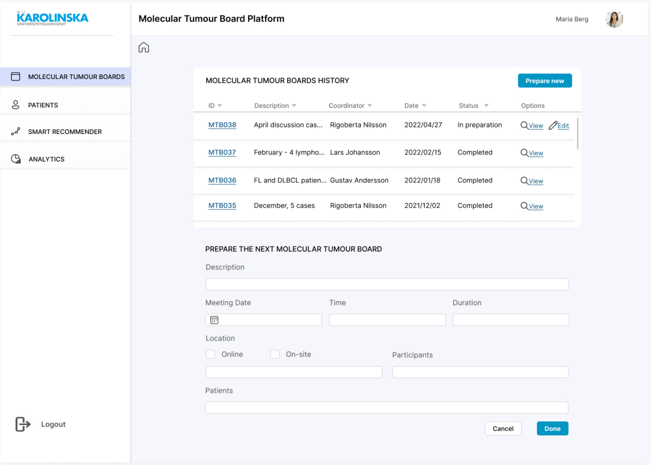

Figure S4. Board preparation.

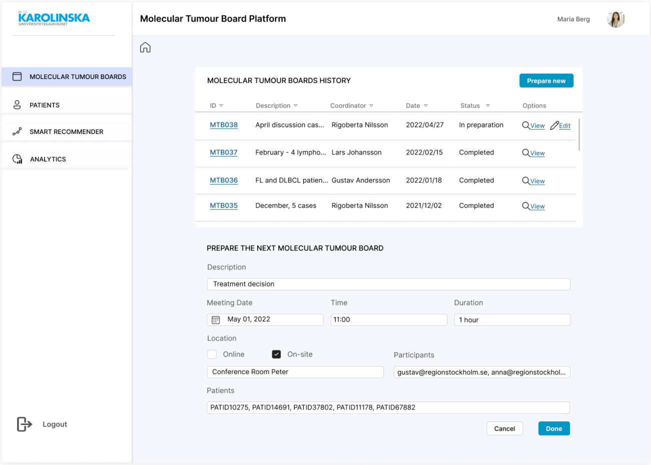

Figure S5. Board preparation 2.

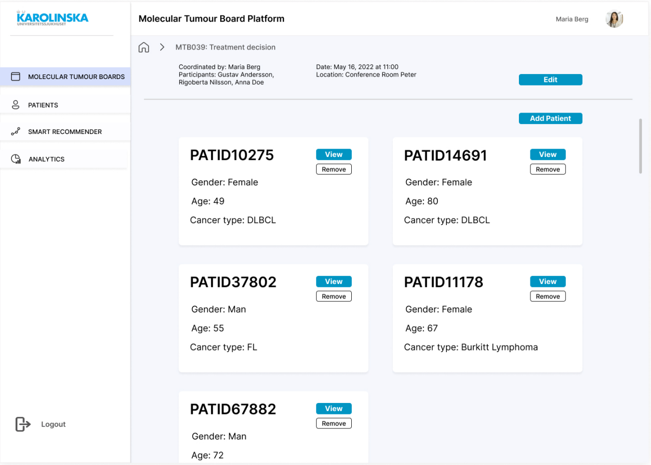

Figure S6. Board overview.

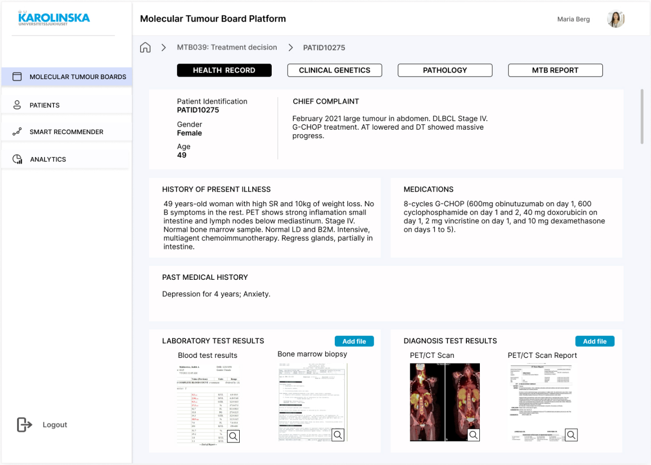

Figure S7. Health record.

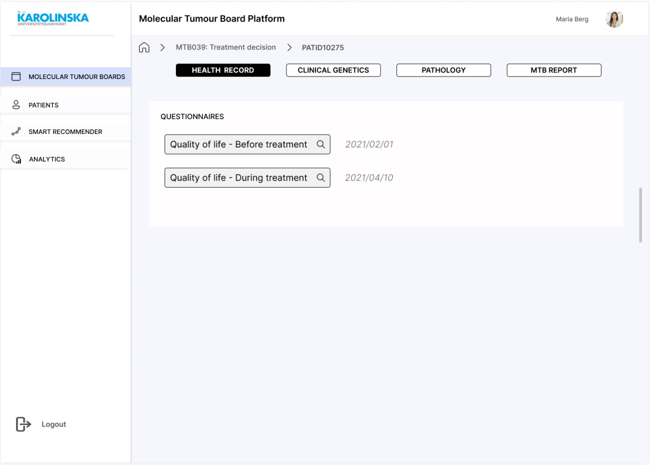

Figure S8. Health record 2.

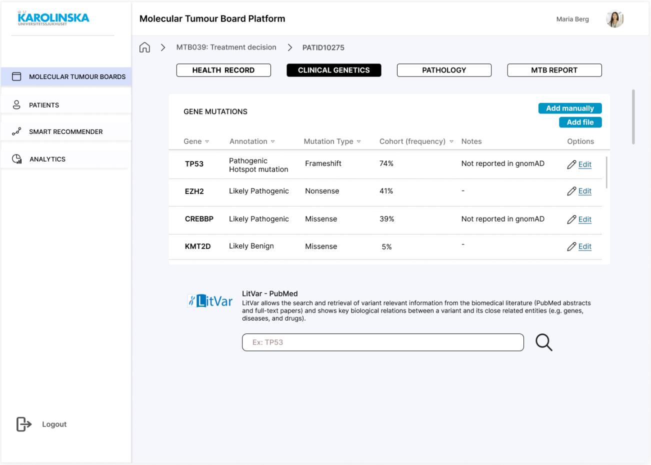

Figure S9. Clinical genetics.

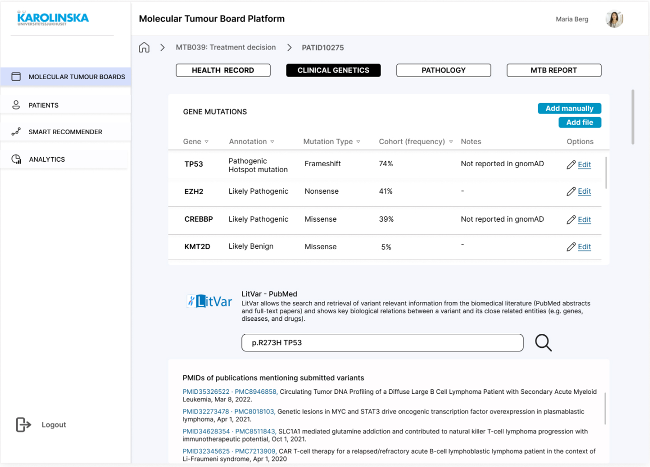

Figure S10. Clinical genetics 2.

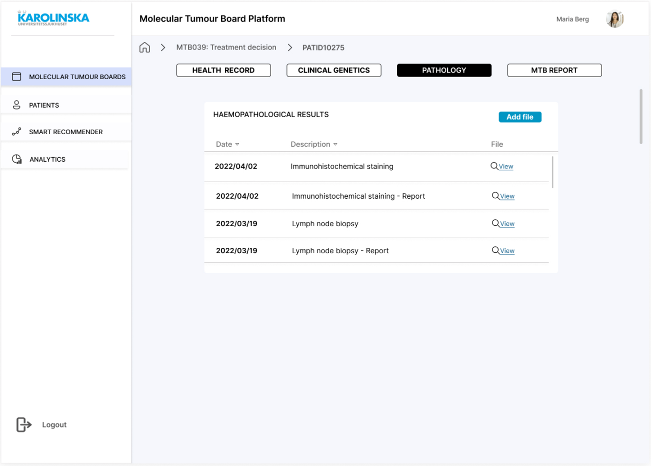

Figure S11. Pathology.

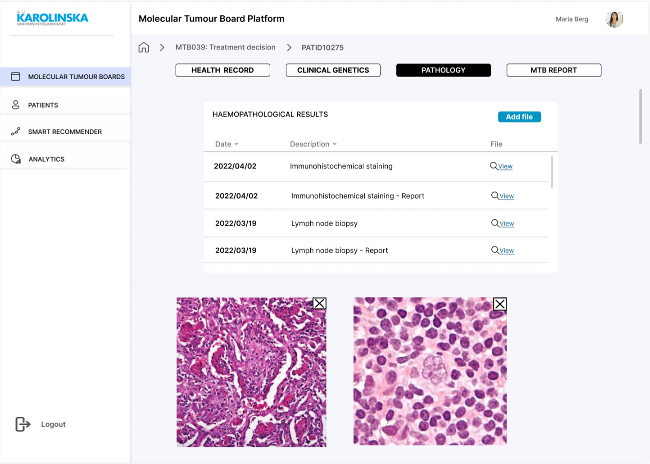

Figure S12. Pathology 2.

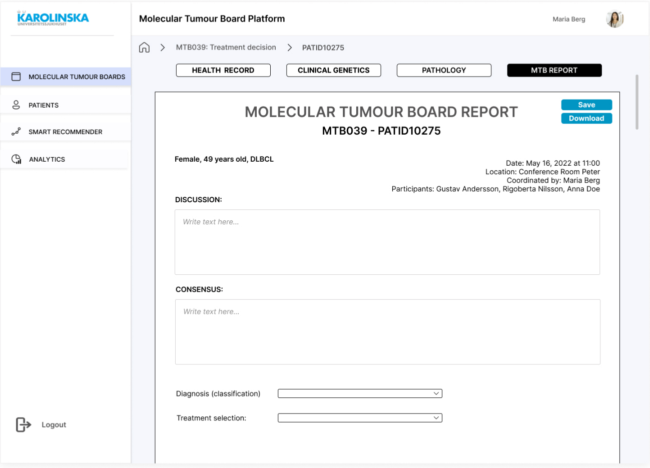

Figure S13. MTB report.

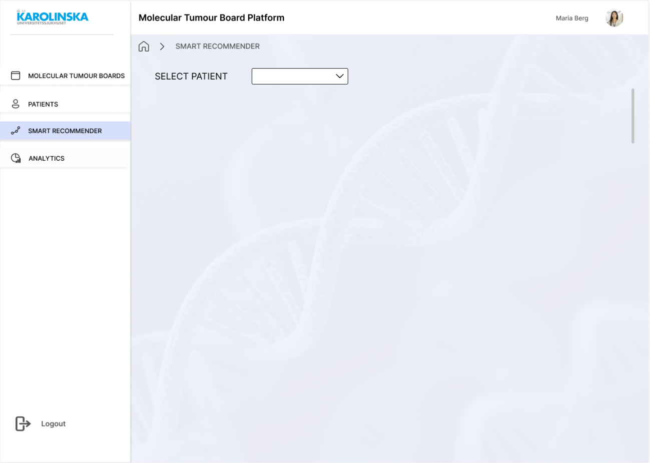

Figure S14. Recommender.

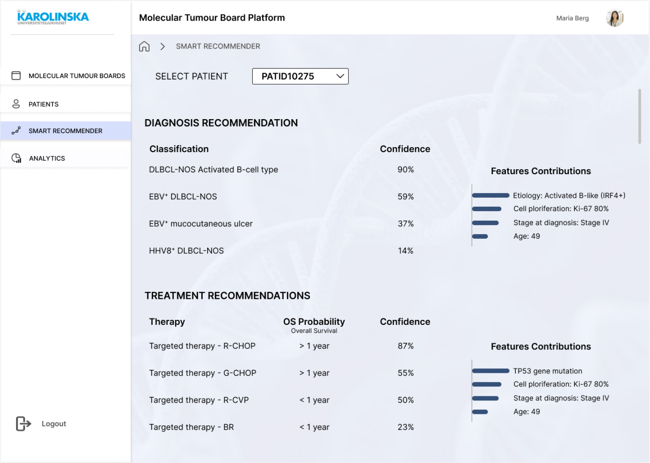

Figure S15. Recommender 2.

Our mock-up demonstration can be accessed at: `https://bit.ly/MTBmockup`

REFERENCES

[1]Singer J, Irmisch A, Ruscheweyh HJ, Singer F, Toussaint NC, Levesque MP, et al. Bioinformatics for precision oncology. *Briefings in Bioinformatics* **20** (2019) 778–788. doi:10.1093/bib/bbx143.

[2]Horak P, Leichsenring J, Goldschmid H, Kreutzfeldt S, Kazdal D, Teleanu V, et al. Assigning evidence to actionability: An introduction to variant interpretation in precision cancer medicine. *Genes, Chromosomes & Cancer* (2021). doi:10.1002/gcc.22987.

[3]Reimer N, Unberath P, Busch H, Börries M, Metzger P, Ustjanzew A, et al. Challenges and Experiences Extending the cBioPortal for Cancer Genomics to a Molecular Tumor Board Platform. *Studies in Health Technology and Informatics* **287** (2021) 139–143. doi:10.3233/SHTI210833.

[4]Buechner P, Hinderer M, Unberath P, Metzger P, Boeker M, Acker T, et al. Requirements Analysis and Specification for a Molecular Tumor Board Platform Based on cBioPortal. *Diagnostics* **10** (2020) 93. doi:10.3390/diagnostics10020093.

[5]Reimer N, Ulrich H, Busch H, Kock-Schoppenhauer AK, Ingenerf J. openEHR Mapper - A Tool to Fuse Clinical and Genomic Data Using the openEHR Standard. *Studies in Health Technology and Informatics* **278** (2021) 86–93. doi:10.3233/SHTI210055.

[6]Macchia G, Ferrandina G, Patarnello S, Autorino R, Masciocchi C, Pisapia V, et al. Multidisciplinary Tumor Board Smart Virtual Assistant in Locally Advanced Cervical Cancer: A Proof of Concept. *Frontiers in Oncology* **11** (2022). doi:10.3389/fonc.2021.797454.

[7]Munoz-Gama J, Martin N, Fernandez-Llatas C, Johnson OA, Sepúlveda M, Helm E, et al. Process mining for healthcare: Characteristics and challenges. *Journal of Biomedical Informatics* **127** (2022) 103994. doi:10.1016/j.jbi.2022.103994.

[8]Walsh S, de Jong EEC, van Timmeren JE, Ibrahim A, Compter I, Peerlings J, et al. Decision Support Systems in Oncology. *JCO clinical cancer informatics* **3** (2019) 1–9. doi:10.1200/CCI.18.00001.

[9]Martínez-García M, Hernández-Lemus E. Data Integration Challenges for Machine Learning in Precision Medicine. *Frontiers in Medicine* **8** (2022). doi:10.3389/fmed.2021.784455.

[10]Hammer RD, Fowler D, Sheets LR, Siadimas A, Guo C, Prime MS. A digital tumor board solution impacts case discussion time and postponement of cases in tumor boards. *Health and Technology* **11** (2021) 525–533. doi:10.1007/s12553-021-00533-x.

[11]Hoffmann M, Vander Stichele R, Bates DW, Björklund J, Alexander S, Andersson ML, et al. Guiding principles for the use of knowledge bases and real-world data in clinical decision support systems: report by an international expert workshop at Karolinska Institutet. *Expert Review of Clinical Pharmacology* **13** (2020) 925–934. doi:10.1080/17512433.2020.1805314.

[12]Blasi L, Bordonaro R, Serretta V, Piazza D, Firenze A, Gebbia V. Virtual Clinical and Precision Medicine Tumor Boards-Cloud-Based Platform-Mediated Implementation of Multidisciplinary Reviews Among Oncology Centers in the COVID-19 Era: Protocol for an Observational Study. *JMIR research protocols* **10** (2021) e26220. doi:10.2196/26220.

[13]Santaolalla A, Hulsen T, Davis J, Ahmed HU, Moore C, Punwani S, et al. The ReIMAGINE Multimodal Warehouse: Using Artificial Intelligence for Accurate Risk Stratification of Prostate Cancer. *Frontiers in Artificial Intelligence* **4** (2021). doi:10.3389/frai.2021.769582.

[14]Pishvaian MJ, Blais EM, Bender RJ, Rao S, Boca SM, Chung V, et al. A virtual molecular tumor board to improve efficiency and scalability of delivering precision oncology to physicians and their patients. *JAMIA Open* **2** (2019) 505–515. doi:10.1093/jamiaopen/ooz045.

[15]Tamborero D, Dienstmann R, Rachid MH, Boekel J, Baird R, Braña I, et al. Support systems to guide clinical decision-making in precision oncology: The Cancer Core Europe Molecular Tumor Board Portal. *Nature Medicine* **26** (2020) 992–994. doi:10.1038/s41591-020-0969-2.

[16]Green MF, Bell JL, Hubbard CB, McCall SJ, McKinney MS, Riedel JE, et al. Implementation of a Molecular Tumor Registry to Support the Adoption of Precision Oncology Within an Academic Medical Center: The Duke University Experience. *JCO precision oncology* **5** (2021) PO.21.00030. doi:10.1200/PO.21.00030.

[17]Borchert F, Mock A, Tomczak A, Hügel J, Alkarkoukly S, Knurr A, et al. Knowledge bases and software support for variant interpretation in precision oncology. *Briefings in Bioinformatics* **22** (2021) bbab134. doi:10.1093/bib/bbab134.

[18]Luchini C, Lawlor RT, Milella M, Scarpa A. Molecular Tumor Boards in Clinical Practice. *Trends in Cancer* **6** (2020) 738–744. doi:10.1016/j.trecan.2020.05.008.

[19]Mammoliti A, Smirnov P, Nakano M, Safikhani Z, Eeles C, Seo H, et al. Orchestrating and sharing large multimodal data for transparent and reproducible research. *Nature Communications* **12** (2021). doi:10.1038/s41467-021-25974-w.

[20]Horgan D, Curigliano G, Rieß O, Hofman P, Büttner R, Conte P, et al. Identifying the Steps Required to Effectively Implement Next-Generation Sequencing in Oncology at a National Level in Europe. *Journal of Personalized Medicine* **12** (2022) 72. doi:10.3390/jpm12010072.

[21]Pang Z, Zhang S, Yang Y, Qi J, Yang P. Interoperable Multi-Modal Data Analysis Platform for Alzheimer's Disease Management. *The 18th IEEE International Symposium on Parallel and Distributed Processing with Applications* (2020). doi:10.1109/ISPA-BDCloud-SocialCom-SustainCom51426.2020.00196.

[22]Schönthaler M, Schlomm T. Network medicine and health services research in urology. *Der Urologe. Ausg. A* **60** (2021) 39–44. doi:10.1007/s00120-020-01406-3.
